# p53-Dependent ENOX2 Downregulation Mediates the Apoptotic Responses to Heteroarene-Fused Anthraquinones in Colon Cancer Cells

**DOI:** 10.3390/biom16071043

**Published:** 2026-07-17

**Authors:** Chien-Yu Chen, Alexander S. Tikhomirov, Yih-Farng Liou, Chi-Wen Chen, Shih-Han Chiu, Atikul Islam, Andrey E. Shchekotikhin, Pin Ju Chueh

**Affiliations:** 1 Graduate Institute of Biomedical Sciences, College of Medicine, National Chung Hsing University, Taichung 402202, Taiwan; z920911419@gmail.com (C.-Y.C.); wmir1429@gmail.com (S.-H.C.); islammiu555@gmail.com (A.I.); 2 Gause Institute of New Antibiotics, 11 B. Pirogovskaya Street, Moscow 119021, Russia; tikhomirov@gause-inst.ru; 3 Department of Internal Medicine, Feng-Yuan Hospital, Ministry of Health and Welfare, Taichung 42055, Taiwan; liouyihfarngdoc8@gmail.com; 4 Department of Surgery, Feng-Yuan Hospital, Ministry of Health and Welfare, Taichung 42055, Taiwan; a0958130438@gmail.com; 5 Graduate Institute of Chinese Medicine and Drug Development, College of Medicine, National Chung Hsing University, Taichung 402202, Taiwan

**Keywords:** anthraquinone, apoptosis, cell cycle arrest, heterocyclic compounds, colon cancer cells, ecto-NADH oxidase disulfide-thiol exchanger 2 (ENOX2/tNOX)

## Abstract

Anthraquinone-based intercalating compounds, such as doxorubicin and mitoxantrone, have long been used clinically due to their ability to induce DNA damage. More recently, heteroarene-fused anthraquinones have been developed to further enhance their anticancer activity. Among these compounds, 4,11-bis(2-(2-chloroacetamidine)ethylamino)anthra[2,3-b]thiophene-5,10-dione dihydrochloride (designated as derivative **a**) was identified as a potent apoptotic inducer. Based on this scaffold, two additional derivatives were synthesized by replacing the sulfur atom within the heterocyclic ring with nitrogen (derivative **b**) or oxygen (derivative **c**). Building upon our previous identification of ENOX2 as the primary target of this scaffold, the present study investigated the antiproliferative effects and underlying mechanisms of these derivatives in colon cancer cells with varying p53 statuses. Derivatives **a** and **b** effectively induced apoptosis and suppressed proliferation in p53 wild-type HCT116 cells, which was concomitantly accompanied by significant ENOX2 downregulation and the activation of intrinsic apoptotic signaling. In contrast, p53-null HCT116 cells exhibited reduced sensitivity, attenuated apoptotic responses, and minimal ENOX2 downregulation. Notably, derivative **c** primarily induced G2/M arrest rather than apoptosis regardless of p53 status, indicating a predominantly cytostatic mechanism. Collectively, these findings suggest that the degree of ENOX2 modulation is linked to the distinct anti-proliferative responses induced by heteroarene-fused anthraquinones, and that p53 status serves as a critical molecular switch influencing the transition between cytostatic growth arrest and apoptotic cell death.

## 1. Introduction

Despite significant advances in cancer therapy, cancer remains one of the leading causes of morbidity and mortality worldwide, emphasizing the need for novel therapeutic strategies with improved efficacy and safety. Conventional chemotherapeutic agents, while effective, often possess a narrow therapeutic index, resulting in off-target toxicity and the emergence of drug resistance. These challenges highlight the importance of identifying cancer-associated molecules that serve as selective and mechanistically actionable drugs.

Anthraquinone-based compounds represent a versatile class of anticancer agents. Although clinically approved drugs such as doxorubicin and mitoxantrone act mainly through DNA intercalation and topoisomerase II inhibition, growing evidence indicates that anthraquinone derivatives can be optimized to selectively target cancer-associated proteins, expanding their therapeutic potential beyond conventional DNA-directed mechanisms [1,2,3,4,5,6,7,8,9,10]. However, the therapeutic utility of these compounds is frequently constrained by severe systemic side effects and the frequent emergence of multidrug resistance. To address these limitations, next-generation anthraquinone derivatives have been developed, including heteroarene-fused analogs that have demonstrated enhanced anticancer potency [11]. In our previous work, we documented the synthesis and anticancer characteristics of 4,11-bis(2-(2-chloroacetamidine)ethylamino)anthra[2,3-b]thiophene-5,10-dione dihydrochloride (derivative **a**) and its two analogs, produced by substituting the sulfur atom in the heteroarene ring with nitrogen (derivative **b**) or oxygen (derivative **c**) [12]. Using molecular docking simulations and cellular thermal shift assays (CETSA), we identified ecto-NADH oxidase disulfide-thiol exchanger 2 (ENOX2/tNOX) as their primary molecular target, with compound exposure leading to the transcriptional and protein-level downregulation of ENOX2 in both p53-functional SAS and p53-mutant HSC-3 oral cancer cells [12].

ENOX2 is a cancer-associated redox enzyme that has emerged as a key regulator of malignant progression. Accumulating evidence implicates ENOX2 in multiple hallmarks of cancer, including sustained proliferation, migration, invasion, cancer stemness, immune evasion, and therapeutic resistance [13,14,15,16,17,18]. Elevated ENOX2 expression has also been associated with aggressive tumor behavior and poor clinical outcomes in several malignancies [19,20,21,22]. These findings position ENOX2 as both clinically relevant biomarker and a promising therapeutic target [20,23,24]. Consistently, diverse natural and synthetic compounds have been reported to suppress malignant phenotypes by directly targeting ENOX2-dependent pathways, leading to apoptosis, senescence, and inhibition of tumor progression [14,24,25,26,27,28]. Recent genetic and epigenetic studies further suggest that the biological functions of ENOX2 extend beyond cancer, implicating this protein in a broader spectrum of human diseases [29,30].

In the present study, we sought to extend our previous observations to a colon cancer model and elucidate whether p53 status dictates the anti-proliferative responses induced by these heteroarene-fused anthraquinones. We report that all three derivatives suppress proliferation in colon cancer cells; however, derivatives **a** and **b** preferentially induce apoptosis in p53 wild-type cells, whereas derivative **c** predominantly triggers cell cycle arrest. In contrast, p53-null cells displayed significantly attenuated apoptotic sensitivity despite retaining partial growth suppression, a phenomenon that likely reflects differences in the efficacy of ENOX2 modulation under different p53 backgrounds.

## 2. Materials and Methods

### 2.1. Chemistry

**Figure 1:**
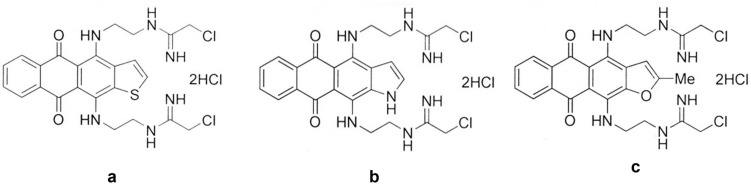
Structures of heteroarene-fused anthraquinone derivatives **a**, **b**, and **c**.

The heteroarene-fused anthraquinone derivatives used in this study were previously designed, synthesized, purified, and fully characterized as described in our earlier report [13]. Their structural characterization, including melting points, ^1^H and ^13^C NMR spectroscopy, HRMS, and HPLC purity analyses, has been reported previously [13]. These compounds share a common anthraquinone scaffold but differ in the fused heteroarene moiety, namely thiophene (derivative **a**), indole (derivative **b**), and furan (derivative **c**), allowing evaluation of the influence of heteroarene substitution on their biological activities. The resulting products—4,11-bis(2-(2-chloroacetamidine)ethylamino)anthra[2,3-b]thiophene-5,10-dione dihydrochloride (designated derivative **a**), 4,11-bis(2-(2-chloroacetamidine)ethylamino)naphtho[2,3-f]indole-5,10-dione dihydrochloride (derivative **b**), and 4,11-bis(2-(2-chloroacetamidine)ethylamino)-2-methylanthra[2,3-b]furan-5,10-dione dihydrochloride (derivative **c**) (Figure 1)—were obtained with a purity greater than 95%, as confirmed by HPLC analysis. Concentrated stock solutions were prepared in DMSO, and fresh working solutions were prepared immediately before each experiment.

### 2.2. Cell Culture and Reagent

Primary antibodies against Bak (Cat. No. 12105, 1:1000), Bax (Cat. No. 2772, 1:1000), Bcl-2 (Cat. No. 15071, 1:1000), and PARP (Cat. No. 9542, 1:1000) were obtained from Cell Signaling Technology, Inc. (Danvers, MA, USA). An anti-β-actin (Cat. No. 66009, 1:40,000) antibody was purchased from Millipore Corp. (Temecula, CA, USA), whereas the anti-ENOX2 (tNOX) antiserum was generated in our laboratory as previously described [31]. Unless otherwise specified, all chemicals and reagents were obtained from Sigma Chemical Company (St. Louis, MO, USA).

HCT116 (human colorectal cancer) wild-type (The Bioresource Collection and Research Center, BCRC, Hsinchu, Taiwan) and p53^−/−^ (from Horizon Discovery, Cambridge, UK) cells were grown in McCoy’s 5A medium. All media were supplemented with 10% FBS, 100 units/mL penicillin, and 50 µg/mL streptomycin. Cells were maintained at 37 °C in a humidified atmosphere of 5% CO_2_ in air, and the media were replaced every 2–3 days. Concentrated stock solutions of the individual derivatives were prepared in DMSO, subsequently diluted with sterile water, and further diluted in culture medium to achieve the indicated final treatment concentrations Control cells received equivalent volumes of DMSO and sterile water (vehicle control). Cells were treated with different concentrations as described in the text. The HCT116 p53 wild-type and p53-null cell lines share the same genetic background, including wild-type APC status, with TP53 deletion representing the principal genetic difference between the two cell lines.

### 2.3. Continuous Monitoring of Cell Growth by Cell Impedance Measurements

For continuous monitoring of changes in cell growth, 7.5 × 10^3^ cells per well were seeded onto E-plates and incubated for 30 min at room temperature before being transferred to the xCELLigence System (Roche, Mannheim, Germany). After overnight incubation, cells were treated with the indicated derivatives or vehicle control, and real-time impedance measurements were recorded at 1-h intervals. Cell growth was monitored as the Cell Index (CI) using the xCELLigence RTCA software version 1.2. The Cell Index for each well was normalized to the last measurement recorded immediately before compound treatment (approximately18 h after cell seeding), which served as the normalization time point. Following normalization, all treatment groups exhibited comparable baseline Cell Index values prior to treatment, indicating consistent cell seeding and attachment across all wells. Each treatment condition was analyzed in duplicate wells (technical replicates), and the experiment was independently performed twice (biological replicates).

### 2.4. Apoptosis Determination

Apoptosis was assessed using an Annexin V-FITC Apoptosis Detection Kit (BD Pharmingen, San Jose, CA, USA). Cells cultured in 6-cm dishes were harvested by trypsinization, collected by centrifugation, and washed once with PBS. The cell pellet was resuspended in 1× binding buffer and stained with Annexin V-FITC, according to the manufacturer’s instructions. Propidium iodide (PI) was added to distinguish necrotic and late apoptotic cells. Early apoptotic cells were defined as Annexin V-FITC-positive/PI-negative, while late apoptotic or necrotic cells were Annexin V-FITC-positive/PI-positive. Apoptosis was quantified as the combined percentage of Annexin V-positive/PI-negative and Annexin V-positive/PI-positive cells, whereas PI-positive/Annexin V-negative cells were considered necrotic and were not included in the apoptotic population. Samples were analyzed on a CytoFLEX LX cytometer operated with CytExpert software, version 2.6.0 (Beckman Coulter Inc., Brea, CA, USA), and apoptosis was quantified as the percentage of Annexin V-positive and/or PI-positive cells. A minimum of 10,000 events were acquired and analyzed for each sample.

### 2.5. Cell Cycle Determination

In brief, after treatments, 10^6^ cells were harvested, washed with PBS, and fixed by the gradual addition of 75% ethanol before storage at −20 °C for at least 1 h. Fixed cells were washed with PBS and centrifuged at 500× *g* for 5 min. The pellet was resuspended in 200 μL ice-cold PBS and incubated with propidium iodide (PI) staining solution (20 mM Tris pH 8.0, 1 mM NaCl, 0.1% NP-40, 1.4 mg/mL RNase A, 0.05 mg/mL PI) for 30 min on ice in the dark. DNA content was subsequently analyzed using a CytoFLEX LX cytometer operated with CytExpert software, version 2.6.0 (Beckman Coulter Inc., Brea, CA, USA).

### 2.6. Cellular Thermal Shift Assays (CETSA) to Evaluate ENOX2 Binding

Direct target engagement of the anthraquinone derivative with ENOX2 was evaluated using the cellular thermal shift assay (CETSA). To evaluate direct target engagement independent of cellular uptake or metabolism, a lysate-based CETSA was performed in which compounds were added directly to cell lysates following cell disruption. Briefly, cells (2 × 10^7^) were plated in 10-cm culture dishes and cultured overnight. Prior to cell harvest, cells were incubated with 10 μM MG132 for 1 h, washed twice with PBS, detached by trypsinization, and collected by centrifugation at 12,000 rpm for 3 min at room temperature. The resulting cell pellets were washed once with PBS by centrifugation at 7500 rpm for 3 min and then resuspended in 1 mL of lysis buffer containing 20 mM Tris-HCl, pH 7.4, 100 mM NaCl, 5mM EDTA, 2 mM phenylmethylsulfonyl fluoride (PMSF), 10 ng/mL leupeptin, and 10 μg/mL aprotinin. Cell lysates were prepared by three consecutive freeze–thaw cycles consisting of immersion in liquid nitrogen for 3 min, incubation at 37 °C for 3 min, and brief vortexing. Following completion of the freeze–thaw lysis procedure, derivative **a** was added directly to the resulting cell lysates at a final concentration of 20 μM, whereas an equal volume of vehicle was added to the corresponding control samples. Following incubation at 37 °C for 1 h, lysates were aliquoted (100 μL per tube) and heated individually at 40 °C, 43 °C, 46 °C, 49 °C, 52 °C, 55 °C, 58 °C, 61 °C, or 67 °C for 3 min. After heat treatment, denatured proteins were removed by centrifugation at 12,000 rpm for 30 min at 4 °C. The soluble fractions were subsequently analyzed by SDS-PAGE followed by Western blotting using antisera to ENOX2/tNOX. β-Actin served as the loading control.

### 2.7. Immunoblot Analysis

Whole-cell lysates were prepared using lysis buffer containing 20 mM Tris-HCl pH 7.4, 100 mM NaCl, 5 mM EDTA, 2 mM phenylmethylsulfonyl fluoride (PMSF), 10 ng/mL leupeptin, and 10 μg/mL aprotinin. Protein concentrations were determined, and equal amounts of protein (40 µg) were separated by SDS-PAGE and electrotransferred onto PVDF membranes (Schleicher & Schuell, Keene, NH, USA). Following transfer, the membranes were briefly stained with Ponceau S to verify protein transfer efficiency and then blocked with 5% non-fat milk in TBST for 1 h at room temperature. The membranes were subsequently incubated overnight at 4 °C with the appropriate primary antibodies. After primary antibody incubation, the membranes were washed three times with TBST (10 min per wash) at room temperature with gentle agitation, followed by incubation with horseradish peroxidase (HRP)-conjugated secondary antibodies for 1 h at room temperature. The membranes were then washed three additional times with TBST (10 min per wash) at room temperature with gentle agitation, followed by incubation with horseradish peroxidase (HRP)-conjugated secondary antibodies for 1 h at room temperature. The membranes were then washed three additional times with TBST (10 min per wash) before immunoreactive bands were visualized using enhanced chemiluminescence (ECL) detection reagents (Amersham Biosciences, Piscataway, NJ, USA) according to the manufacturer’s instructions. The resulting chemiluminescent films were scanned to generate digital images, and band intensities were quantified by densitometric analysis using ImageJ software version 1.54 (National Institutes of Health, Bethesda, MD, USA). The relative expression of each target protein was calculated by normalizing its band intensity to the corresponding β-actin band (target protein/β-actin ratio) and expressed as fold changes relative to the control group.

### 2.8. Statistics

All data are expressed as the means ± SE of three independent experiments. The significance of differences between control and treatment groups was calculated using a one-way ANOVA.

## 3. Results

### 3.1. Heteroarene-Fused Anthraquinones Suppress Colon Cancer Cell Proliferation via Apoptosis and Cell Cycle Arrest in p53 Wild-Type HCT116 Cells

**Figure 2:**
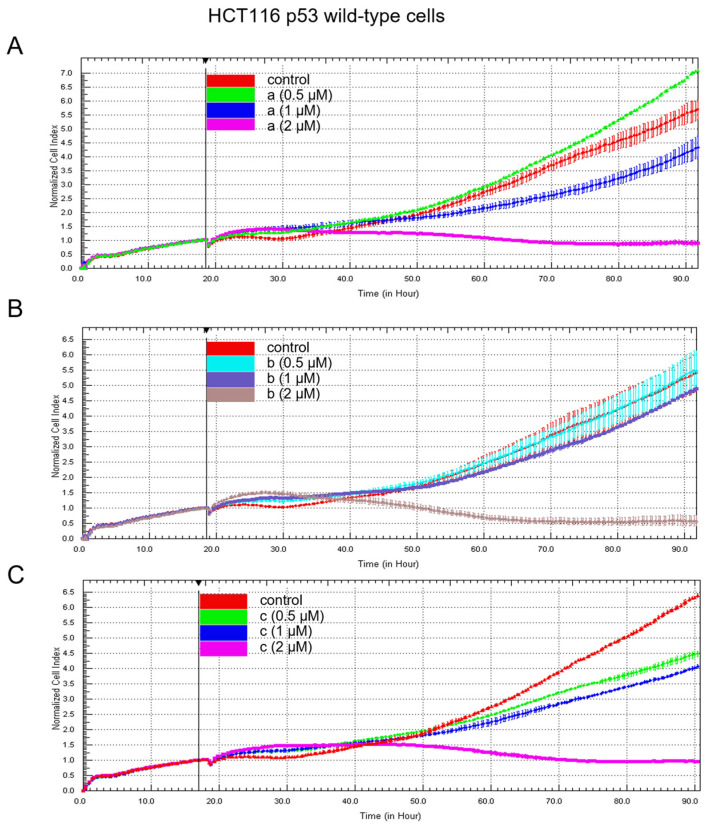
Real-time monitoring of the effects of heteroarene-fused anthraquinone derivatives **a**–**c** on HCT116 p53 wild-type cell proliferation. Cell proliferation was dynamically monitored by impedance measurements in HCT116 p53 wild-type cells, as described in the Materials and Methods. Cells were seeded onto E-plates and allowed to attach overnight prior to treatment (indicated by the vertical line) with increasing concentrations (0.5, 1, and 2 μM) of derivative **a** (**A**), derivative **b** (**B**), or derivative **c** (**C**). Shown are the normalized cell index values measured continuously for up to 90 h following treatment.

In our previous study, we demonstrated the potent anti-proliferative activity of three heteroarene-fused anthraquinones against oral cancer cells [12]. To evaluate whether these effects are conserved in other cancer types, we extended this investigation to a colon cancer model. Real-time cell proliferation was monitored using the xCELLigence RTCA system, and cell index (CI) values were continuously recorded over 90 h to assess the impact of these derivatives on colon cancer cell growth. HCT116 p53 wild-type cells were treated with increasing concentrations (0.5, 1, and 2 μM) of heteroarene-fused anthraquinones **a**–**c**, and growth kinetics were compared with untreated controls (Figure 2). Under control conditions, cells exhibited a steady increase in CI over time, reflecting continuous proliferation. In contrast, treatment with all three derivatives resulted in a robust, concentration-dependent suppression of cell growth. Notably, at 2 μM, each compound markedly arrested proliferation, with CI values plateauing shortly after treatment and subsequently declining, suggesting a transition from growth inhibition to active cytotoxicity. At lower concentrations (0.5 and 1 μM), the derivatives exerted partial yet noticeable inhibitory effects. Although treated cells initially continued to proliferate, their growth rates were distinctly reduced compared to controls, particularly at later time points (>50 h). This trend was consistently observed for derivatives **a** and **c**, whereas derivative **b** exhibited a relatively attenuated inhibitory effect at these lower concentrations. The delayed divergence in growth kinetics suggests a time-dependent impairment of proliferative capacity rather than an immediate cytotoxic response at lower sub-micromolar doses.

**Figure 3:**
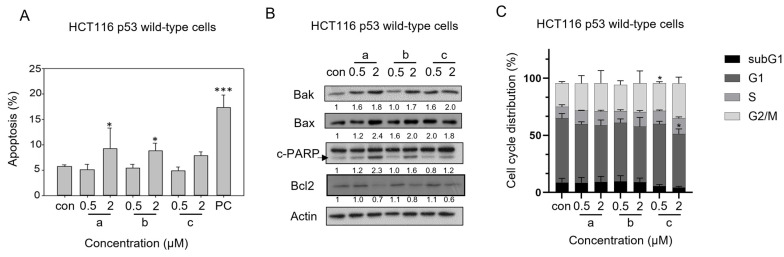
Effects of heteroarene-fused anthraquinone derivatives **a**–**c** on apoptosis and cell cycle progression in HCT116 p53 wild-type cells. (**A**) Analysis of apoptosis by Annexin V-FITC/PI staining following treatment with derivatives **a**, **b**, or **c** at the indicated concentrations (0.5 and 2 μM). H_2_O_2_ treatment was included as a positive control (PC). Values are presented as mean ± SD from three independent experiments (*n* = 3). Statistical significance was determined by one-way ANOVA followed by an appropriate post hoc test. * *p* < 0.05, *** *p* < 0.001 versus control. (**B**) Cells were treated with derivatives **a**, **b**, or **c** at the indicated concentrations for 24 h, and protein expression of apoptosis-related markers was analyzed by Western blotting. Aliquots of cell lysates were resolved by SDS-PAGE and immunoblotted with antibodies against Bak, Bax, cleaved caspase-9, and Bcl-2. β-Actin was used as a loading control. Representative blots are shown. (**C**) Cell cycle distribution was analyzed by flow cytometry following PI staining after treatment with derivatives **a**, **b**, or **c**. Percentages of cells in sub-G1, G1, S, and G2/M phases are shown as stacked bars. Values represent mean ± SD from three independent experiments. Statistical significance was determined by one-way ANOVA followed by an appropriate post hoc test. * *p* < 0.05 versus control. Original Western Blots images are found in Supplementary Materials.

To further investigate the molecular basis of these growth-inhibitory effects, we examined the capability of these compounds to induce apoptosis. Flow cytometric analysis revealed that derivatives **a** and **b** significantly increased apoptotic cell populations at 2 μM (Figure 3A). This apoptotic response was further supported by protein expression profiling via Western blot analysis following treatment with each compound at 2 μM (Figure 3B). Consistent with the observed cytotoxicity effects, treatment with derivatives **a** and **b** resulted in the upregulation of pro-apoptotic proteins Bak and Bax, confirming the activation of the intrinsic mitochondrial apoptotic pathway. This was further corroborated by the presence of cleaved PARP, a hallmark of terminal apoptosis, in cells treated with derivatives **a** and **b,** but not derivative **c**. Interestingly, downregulation of the anti-apoptotic protein Bcl-2 was observed across all three treatment groups, suggesting that the suppression of survival signaling per se is not sufficient to trigger apoptosis in all contexts, and that additional regulatory checkpoints dictate cell fate decisions. This was particularly evident in derivative **c**-treated cells, where apoptosis was not prominently induced, indicating the involvement of alternative anti-proliferative mechanisms. To elucidate these mechanisms of growth inhibition, cell cycle distribution was analyzed by propidium iodide (PI) staining. The results demonstrated that derivative c induced significant G2/M phase arrest at 0.5 μM (Figure 3C). Interestingly, at the higher concentration of 2 μM, the G1 peak was notably reduced and the G2/M arrest became less pronounced compared to the 0.5 μM treatment. This shift, occurring alongside a near-complete plateau in the CI (Figure 2C), suggests that derivative c at higher concentrations induces a more comprehensive cell cycle blockade or an accumulation of cells in the S-phase. Importantly, our apoptosis assays (Figure 3A,B) confirmed that this dramatic reduction in the G1 population was not due to apoptotic cell death. Rather, derivative **c** appears to exert a potent cytostatic effect that sequesters cells within the progression of the cell cycle—likely at the S and G2/M phase—effectively halting population growth without triggering an apoptotic exit. Collectively, these findings indicate that while derivatives a and b act through a p53-associated apoptosis, derivative **c** suppresses proliferation through a predominantly cytostatic, not apoptotic, cell cycle stasis.

### 3.2. The Apoptotic Activity of Heteroarene-Fused Anthraquinones Is Abolished in HCT116 p53-null Cells

**Figure 4:**
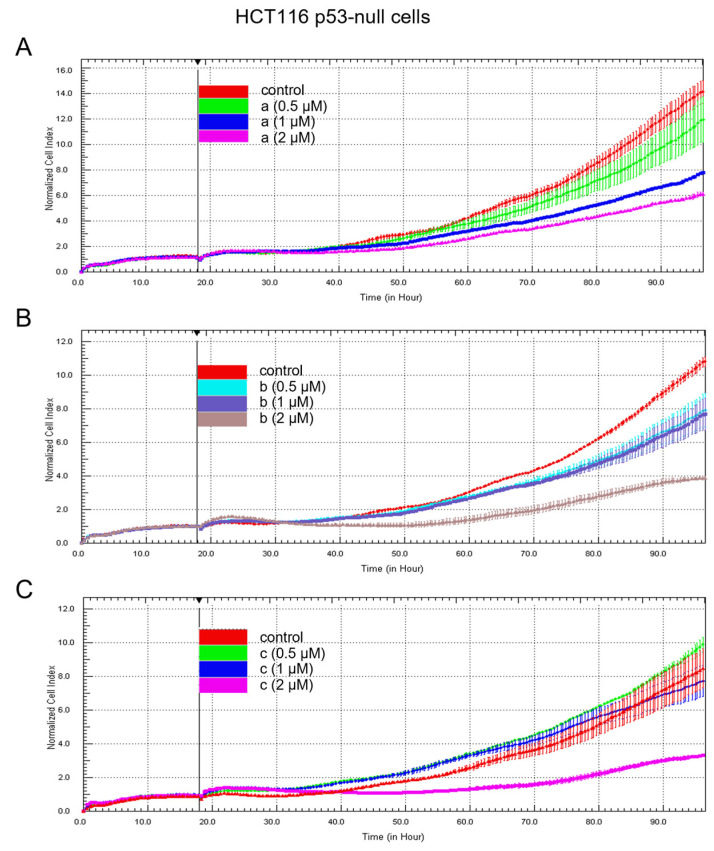
Real-time monitoring of the effects of heteroarene-fused anthraquinone derivatives **a**–**c** on HCT116 p53-null cell proliferation. Cell proliferation was dynamically monitored by impedance measurements in HCT116 p53-null cells, as described in the Materials and Methods. Cells were seeded onto E-plates and allowed to attach overnight prior to treatment (indicated by the vertical line) with increasing concentrations (0.5, 1, and 2 μM) of derivative **a** (**A**), derivative **b** (**B**), or derivative **c** (**C**). Shown are the normalized cell index values measured continuously for up to 90 h following treatment.

To determine whether the growth-inhibitory effects of these analogs are dependent on p53 status, we evaluated their effects in HCT116 p53-null cells (Figure 4). Similar to the observations in p53 wild-type cells, all three derivatives suppressed cell proliferation in a concentration-dependent manner; however, the overall extent of inhibition was markedly attenuated in the absence of p53. Among the compounds, derivative a retained the most consistent dose-dependent inhibitory effect, whereas derivative c showed comparatively diminished activity at lower concentrations, with appreciable suppression observed primarily at 2 μM. Notably, even at the highest concentration, treated cells maintained a gradual increase in CI, indicating that the response in the absence of p53 is predominantly cytostatic, with minimal evidence of apoptotic or overt cytotoxic responses.

**Figure 5:**
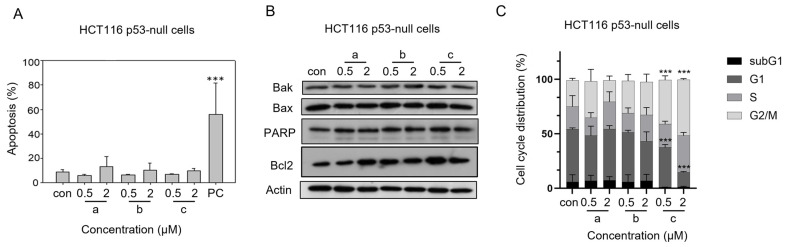
Effects of heteroarene-fused anthraquinone derivatives **a**–**c** on apoptosis and cell cycle progression in HCT116 p53-null cells. (**A**) Apoptotic cell death was determined by Annexin V-FITC/PI staining following treatment with derivatives **a**, **b**, or **c** at the indicated concentrations (0.5 and 2 μM). H_2_O_2_ was included as a positive control (PC). Values are presented as mean ± SD from three independent experiments (*n* = 3). Statistical significance was determined by one-way ANOVA followed by an appropriate post hoc test. *** *p* < 0.001 versus control. (**B**) Cells were treated with derivatives a, b, or c at the indicated concentrations for 24 h, and the expression of apoptosis-related proteins was analyzed by Western blotting. Aliquots of cell lysates were resolved by SDS-PAGE and immunoblotted with antibodies against Bak, Bax, cleaved caspase-9, and Bcl-2. β-Actin was used as a loading control. Representative blots are shown. (**C**) Cell cycle distribution was analyzed by flow cytometry following PI staining after treatment with derivatives **a**, **b**, or **c**. The percentages of cells in sub-G1, G1, S, and G2/M phases are presented as stacked bars. Values represent mean ± SD from three independent experiments. Statistical significance was determined by one-way ANOVA followed by an appropriate post hoc test. *** *p* < 0.001 versus control.

In contrast to p53 wild-type cells, none of the derivatives induced apoptosis in HCT116 p53-null cells (Figure 5A), which was further supported by the lack of activation of key apoptosis-associated proteins, including Bak and Bax (Figure 5B). These findings indicate that the apoptotic signaling triggered by these compounds is strictly dependent on functional p53. Interestingly, cell cycle analysis revealed that only derivative c induced significant G2/M phase arrest at both 0.5 and 2 μM, whereas derivatives **a** and **b** failed to elicit a comparable cell cycle response (Figure 5C). This selective cell cycle modulation suggests that, in the absence of p53, growth suppression is mediated through alternative, compound-specific mechanisms rather than a unified apoptotic program. Collectively, these findings demonstrate that while anti-proliferative effects are partially retained in p53-deficient cells, the ability to engage apoptotic pathways is effectively abolished, underscoring a critical role for p53 in mediating cell death induced by this scaffold. Moreover, the emergence of compound-dependent cell cycle effects highlights a mechanistic divergence under p53-deficient conditions, with derivative **c** preferentially inducing cell cycle arrest as a compensatory anti-proliferative response.

### 3.3. ENOX2 Downregulation Mediates Anti-Proliferative Activity in a p53-Dependent Manner

**Figure 6:**
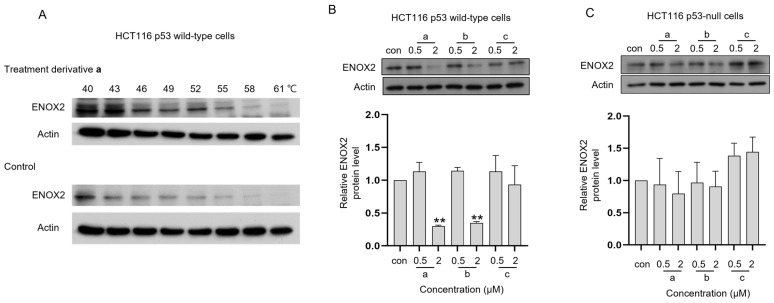
Direct target engagement of ENOX2 by heteroarene-fused anthraquinone derivative **a** revealed by CETSA, and differential regulation of ENOX2 expression by structurally related analogs in HCT116 p53 wild-type and p53-null cells. (**A**) CETSA analysis of ENOX2 thermal stability in HCT116 p53 wild-type cells in the presence and absence of derivative **a**, as described in the Materials and Methods. Following heat treatment, aliquots of cell lysates were resolved by SDS-PAGE and analyzed by Western blotting. β-actin was used as an internal loading control. Representative images are shown. (**B**,**C**) HCT116 p53 wild-type (**B**) or p53-null (**C**) cells were treated with derivatives **a**, **b**, or **c** at the indicated concentrations (0.5 and 2 μM) for 24 h. ENOX2 protein expression was analyzed by Western blot and quantified by densitometric analysis. ENOX2 levels were normalized to β-actin and expressed relative to the untreated control. β-Actin was used as a loading control. Values represent mean ± SD from three independent experiments (*n* = 3). Statistical significance was determined by one-way ANOVA followed by an appropriate post hoc test. ** *p* < 0.01 versus control.

Using molecular docking simulations and CETSA, we previously identified ENOX2 as a direct molecular target of these derivatives, with compound exposure leading to its downregulation in oral cancer cells [12]. Given the observed divergence in apoptotic responses between p53 wild-type and p53-null cells, we next sought to determine whether ENOX2 modulation underlies these context-dependent cellular outcomes. To verify direct binding of derivative **a** to ENOX2 in this model, we performed a lysate-based CETSA to assess whether ENOX2 directly binds derivative **a**. This assay is based on the principle that ligand binding enhances the thermal stability of target proteins, thereby protecting them from heat-induced denaturation [32]. Given our previous demonstration that all three derivatives directly bind ENOX2 in oral cancer cells using the lysate-based CETSA approach [12], we selected derivative **a** as a representative compound to confirm direct binding in the colon cancer context. Our results demonstrated that treatment with derivative **a** increased the thermal stability of ENOX2 (Figure 6A), supporting direct binding of derivative **a** to ENOX2 in HCT116 cell lysates.

Despite this confirmed target engagement, the downstream biological consequences of ENOX2 modulation appeared to be governed by p53 status. Upon treatment, derivatives **a** and **b**, but not derivative **c**, markedly downregulated ENOX2 protein expression in p53 wild-type cells (Figure 6B), which closely correlated with their robust induction of apoptosis (Figure 3A). In contrast, ENOX2 expression was not significantly downregulated in p53-null cells following treatment (Figure 6C), correlating with the total absence of apoptotic induction (Figure 5A). These findings suggest that direct ENOX2 binding alone is insufficient for apoptotic induction. Instead, apoptotic responses were observed only under conditions where ENOX2 protein levels were effectively downregulated, an effect primarily restricted to p53 wild-type cells. In contrast, in p53-null cells, the failure to downregulate ENOX2 was associated with an absence of apoptosis, despite continued suppression of cell proliferation. Importantly, the extent of ENOX2 modulation also appeared to be compound-dependent: derivatives a and b effectively downregulated ENOX2 and induced apoptosis in p53 wild-type cells, whereas derivative c did not significantly reduce ENOX2 levels, indicating a distinct mode of action centered on cell cycle regulation rather than degradation.

## 4. Discussion

Emerging evidence positions ENOX2 as a multifunctional and clinically relevant regulator in cancer, broadening its functional repertoire beyond traditional redox metabolism to include tumor progression, metastasis, and therapeutic response [13,19,20,22,24,33,34]. As a central regulator of intracellular redox balance and NAD^+^ homeostasis, ENOX2 modulates NAD^+^-dependent signaling pathways such as SIRT1. Through the ENOX2–NAD^+^–SIRT1 signaling axis, alterations in cellular redox homeostasis have been proposed to influence p53 acetylation and transcriptional activity, thereby regulating cell fate decisions including proliferation, apoptosis, autophagy, and stress adaptation. Although p53 acetylation was not investigated in the present study, it represents a plausible mechanistic link between ENOX2 downregulation and the p53-dependent apoptotic responses observed following treatment with heteroarene-fused anthraquinones and warrants further investigation. Increasing evidence further suggests that ENOX2 supports malignant phenotypes by promoting metabolic adaptation and pro-survival signaling, while its suppression has emerged as a pivotal anti-cancer event across multiple tumor models. Consistently, pharmacological or genetic downregulation of ENOX2 has been associated with impaired proliferation, disruption of redox homeostasis, attenuation of SIRT1-associated survival pathways, and the induction of apoptosis, autophagy, senescence, or growth arrest across diverse cancer types [27,35]. The present study extends this model in colon cancer cells by demonstrating that heteroarene-fused anthraquinones directly engage ENOX2, as confirmed by CETSA; however, we highlight that target engagement alone appears insufficient to drive apoptosis. Instead, our findings suggest that the magnitude of ENOX2 protein depletion, rather than physical binding alone, more closely correlates with apoptotic responsiveness.

In p53 wild-type HCT116 cells, derivatives **a** and **b** markedly reduced ENOX2 expression, which correlated with the robust activation of intrinsic apoptotic pathways, including the upregulation of Bax/Bak, activation of caspase-9, and resultant PARP cleavage. These findings are consistent with previous reports demonstrating that ENOX2 downregulation is intimately associated with p53-dependent apoptotic signaling under various stresses in other cancer types [27,35]. In contrast, in p53-null cells, ENOX2 expression was not effectively suppressed, and apoptotic induction was abolished, aligning with prior studies suggesting a p53-requisite mechanism [35]. Importantly, both the HCT116 p53 wild-type and isogenic TP53-null cell lines retain wild-type APC, indicating that the differential responses observed in this study are primarily attributable to p53 status rather than differences in APC. Notably, the absence of ENOX2 downregulation in p53-null or mutant contexts is not universal. ENOX2 suppression has been observed in several p53-deficient models, including Hep3B hepatocellular carcinoma and HSC-3 oral cancer cells, where ENOX2 downregulation remained associated with anti-proliferative or cell death responses [27]. Collectively, these observations suggest that although p53 status may influence the downstream cellular responses elicited following ENOX2 suppression, ENOX2 downregulation itself is not strictly dictated by p53 status across all cell types. Rather, suppression of ENOX2 appears to represent a common upstream anti-cancer event, whereas the ultimate cellular outcomes—including apoptosis, autophagy, or cytostatic growth inhibition—are likely shaped by the cell-specific genetic, metabolic, and signaling context.

The precise mechanism underlying ENOX2 downregulation remains to be fully defined. We previously verified that the downregulation of ENOX2 induced by these specific heteroarene-fused anthraquinones was effectively abrogated by the proteasome inhibitor MG132 [12], providing direct evidence that these compounds trigger a proteasome-mediated degradation pathway. This is consistent with other reports demonstrating that pharmacological interventions can reduce ENOX2 protein expression through post-translational mechanisms involving either proteasomal and/or autophagy–lysosomal degradation pathways, depending on the compound and cellular context [27]. The present findings further support the notion that the modulation of protein stability is a critical determinant of anti-cancer responses. Specifically, the requirement for functional p53 to achieve significant ENOX2 downregulation in HCT116 cells suggests that p53 may facilitate the recruitment or activation of the specific ubiquitin-proteasome system (UPS) components—potentially a p53-regulated E3 ubiquitin ligase—necessary for efficient ENOX2 turnover.

The compound-specific differences observed in this study are further elucidated by our previously reported molecular docking simulations [12]. Analysis of the binding modes between these derivatives and the ENOX2 structure revealed that while all three compounds occupy the same binding pocket, they exhibit a clear hierarchy in predicted docking energies: derivative **a** (−128.1 kJ/mol) > derivative **b** (−124.9 kJ/mol) > derivative **c** (−118.7 kJ/mol) [12]. Consistent with our biological observations, derivative **c** was predicted to have the lowest affinity for the ENOX2 target. While all three derivatives interacted with a core set of residues, the unique orientation of derivative **c** and its weaker binding energy appear to be critical factors in its distinct cellular impact. Derivative **c** consistently failed to induce apoptosis, despite retaining anti-proliferative activity. Instead, it preferentially induced G2/M phase arrest at sub-maximal concentrations (0.5 μM) in both p53 wild-type and p53-null cells. At the higher concentration of 2 μM, the reduction in the G1 population without an accompanying increase in apoptotic markers—coinciding with a near-complete plateau in CI—indicates that derivative **c** induces a prolonged, non-apoptotic quiescent state. This suggests a shift toward a more global cell cycle blockade or S-phase trapping at higher doses. Notably, derivative **c** did not significantly downregulate ENOX2 expression, suggesting that a failure to trigger ENOX2 protein reduction may restrict its ability to engage apoptotic pathways. Importantly, these findings emphasize the need to distinguish between the suppression of proliferation and the induction of apoptosis, as these represent fundamentally distinct cellular outcomes. While all three derivatives inhibited cell growth in both p53 wild-type and p53-null cells, this effect was predominantly cytostatic in the absence of p53. Growth inhibition can arise from mechanisms such as cell cycle arrest or metabolic stress without activation of cell death pathways. In contrast, apoptosis is an active and highly coordinated process involving mitochondrial dysfunction and caspase activation. Furthermore, although the xCELLigence assay enabled continuous, real-time monitoring of the dynamic cellular responses following compound treatment, the assay was limited to approximately 90 h; longer-term endpoint assays would provide complementary validation of the sustained antiproliferative effects of these compounds. Our findings therefore suggest that although ENOX2 targeting can impair proliferative capacity independently of p53, the critical transition from cytostasis to apoptosis is strongly influenced by intact p53 signaling.

Several limitations of the present study should be acknowledged. First, although our findings demonstrate a strong association between ENOX2 downregulation and apoptotic induction in p53 wild-type cells, a definitive causal link remains to be established. Rescue experiments, genetic modulation of ENOX2 expression, and comprehensive validation of downstream NAD^+^–SIRT1 signaling were not performed in the current study and will be important for further mechanistic clarification. In addition, while CETSA confirmed compound engagement with ENOX2, the precise molecular mechanisms responsible for p53-dependent turnover remain to be identified. Future studies focusing on post-translational regulation, including proteasomal and autophagy–lysosomal pathways as well as potential E3 ubiquitin ligases involved in ENOX2 turnover, may provide further insight into the regulation of ENOX2 stability in cancer cells. Furthermore, the present study was conducted exclusively in the well-established isogenic HCT116 p53 wild-type and p53-null colorectal cancer cell models. Although this system enabled us to specifically investigate the contribution of p53 while minimizing confounding genetic differences, future studies in additional cancer types, including lung cancer cell lines with defined p53 status, will be important to determine the broader applicability of these findings. Despite these limitations, the present study identifies ENOX2 as an important mediator associated with differential cellular responses to heteroarene-fused anthraquinones and highlights the functional importance of p53 status in governing the transition from cytostatic growth suppression to apoptotic cell death.

## 5. Conclusions

Our data supports a “dual-threshold model” for ENOX2 targeting: a lower threshold where target binding leads to enzymatic inhibition and resultant cell cycle stasis (as seen with derivative **c**), and a higher threshold—dependent on p53—where protein downregulation triggers an irreversible commitment to apoptosis (derivatives **a** and **b**). This indicates that the therapeutic efficacy of heteroarene-fused anthraquinones is determined not only by target engagement but by the specific ability to trigger the degradation of the ENOX2 protein scaffold itself, a process that represents a novel mechanism for overcoming apoptotic resistance.

## Data Availability

The original contributions presented in this study are included in the article and Supplementary Materials. Additional raw data supporting the findings of this study are available from the corresponding author upon reasonable request.

## Supplementary Materials

The following supporting information can be downloaded at: https://www.mdpi.com/article/10.3390/biom16071043/s1.

## Abbreviations

CETSA	Cellular thermal shift assay
CI	Cell indexs
ENOX2	ecto-NADH oxidase disulfide-thiol exchanger 2, Tumor-associated NADH oxidase, tNOX
NAD	Nicotinamide adenine dinucleotide
PI	Propidium iodide
RTCA	Real-time cell analysis
SIRT1	Sirtuin 1
