# Supplementary material for: p53-Dependent ENOX2 Downregulation Mediates the Apoptotic Responses to Heteroarene-Fused Anthraquinones in Colon Cancer Cells
Source: Biomolecules. 2026 Jul 17;16(7):1043. doi: 10.3390/biom16071043 (PMC13407073; doi:10.3390/biom16071043)

Figure 3A Apoptosis HCT116 p53 wild-type cells

Con (H2O)

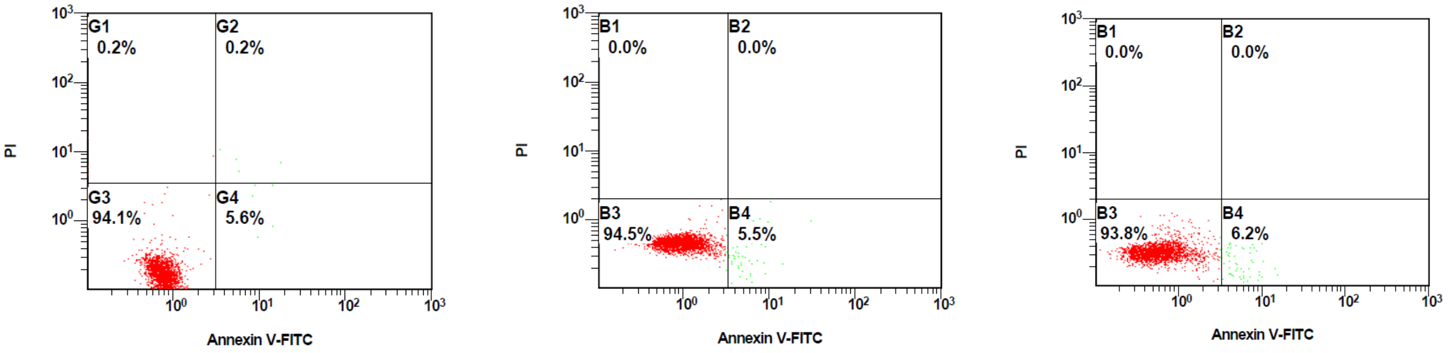

PC

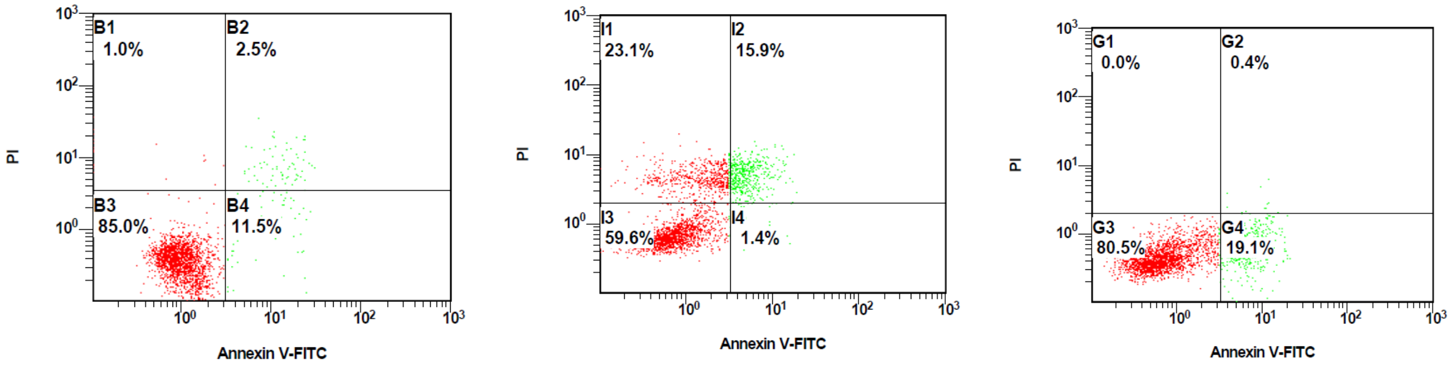

a (0.5μM)

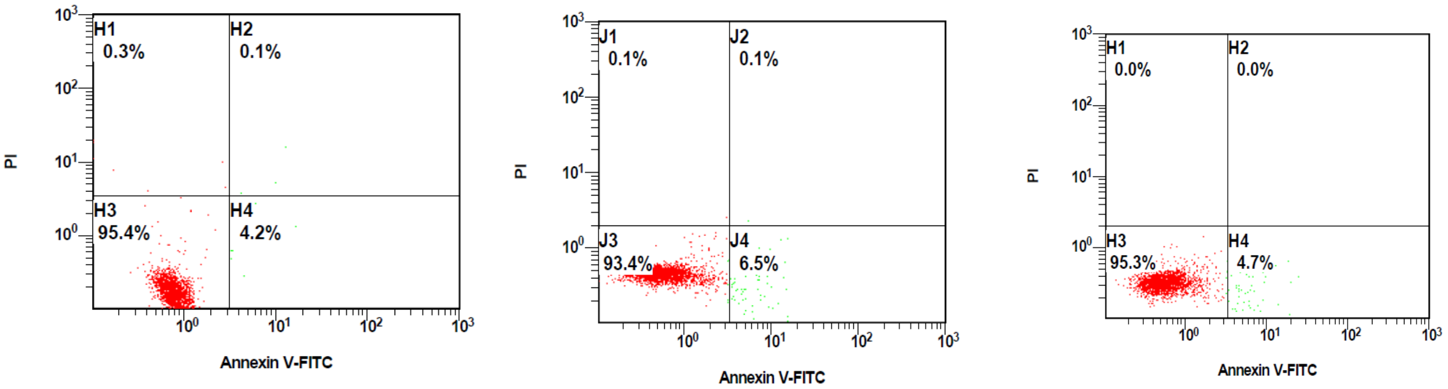

Figure 3A Apoptosis HCT116 p53 wild-type cells

a (2µM)

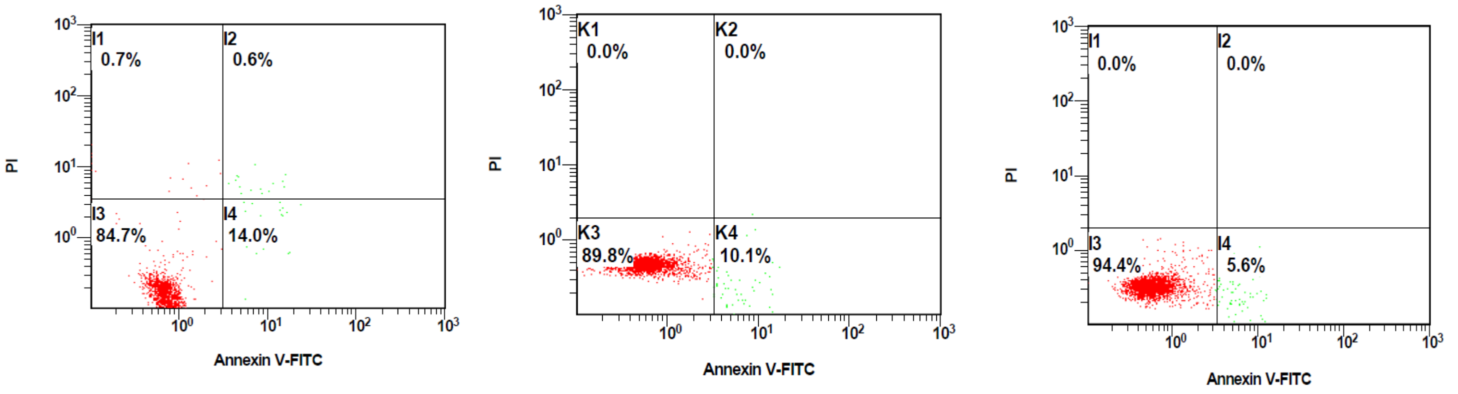

b (0.5µM)

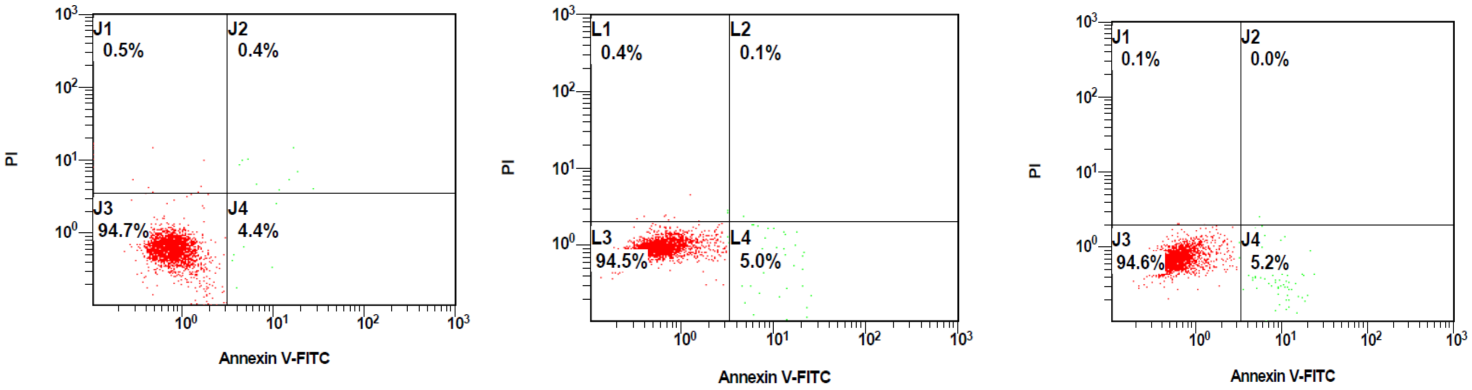

b (2µM)

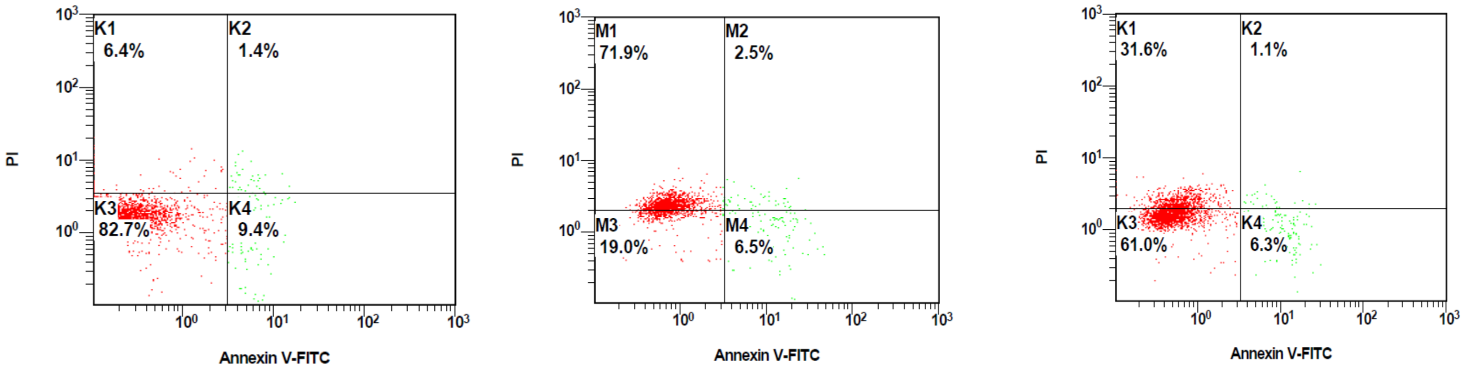

Figure 3A Apoptosis HCT116 p53 wild-type cells

c (0.5µM)

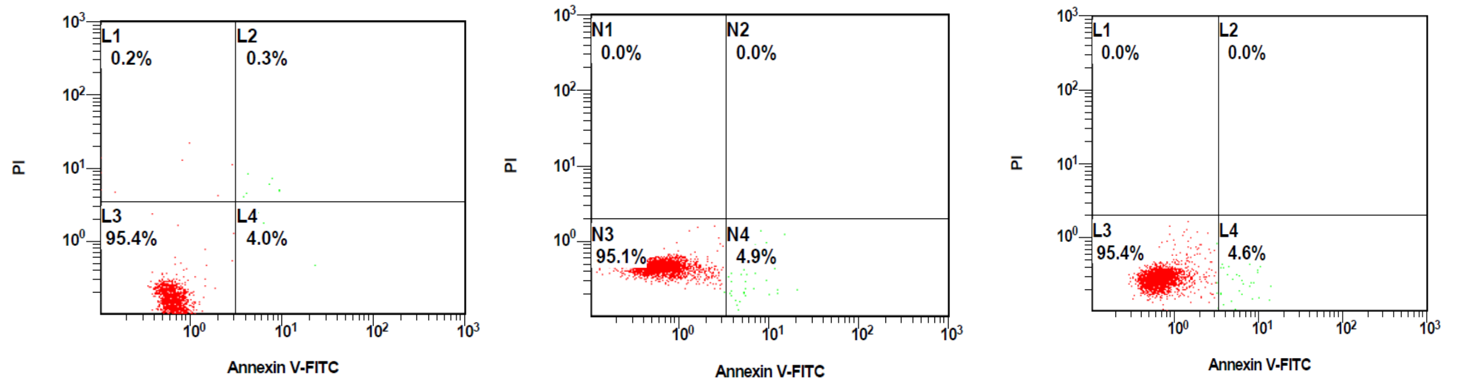

c (2µM)

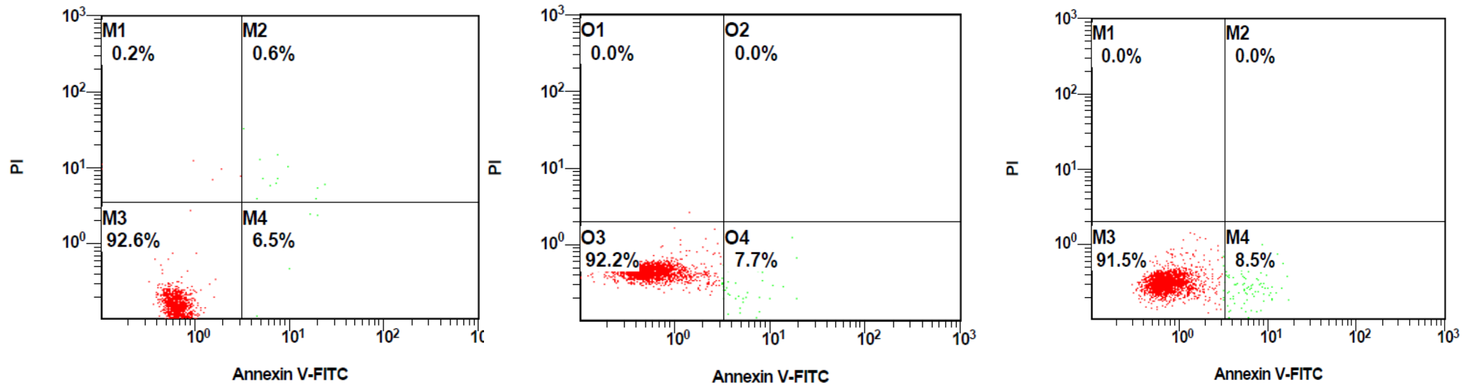

Figure 3B Western blot HCT116 p53 wild-type cells

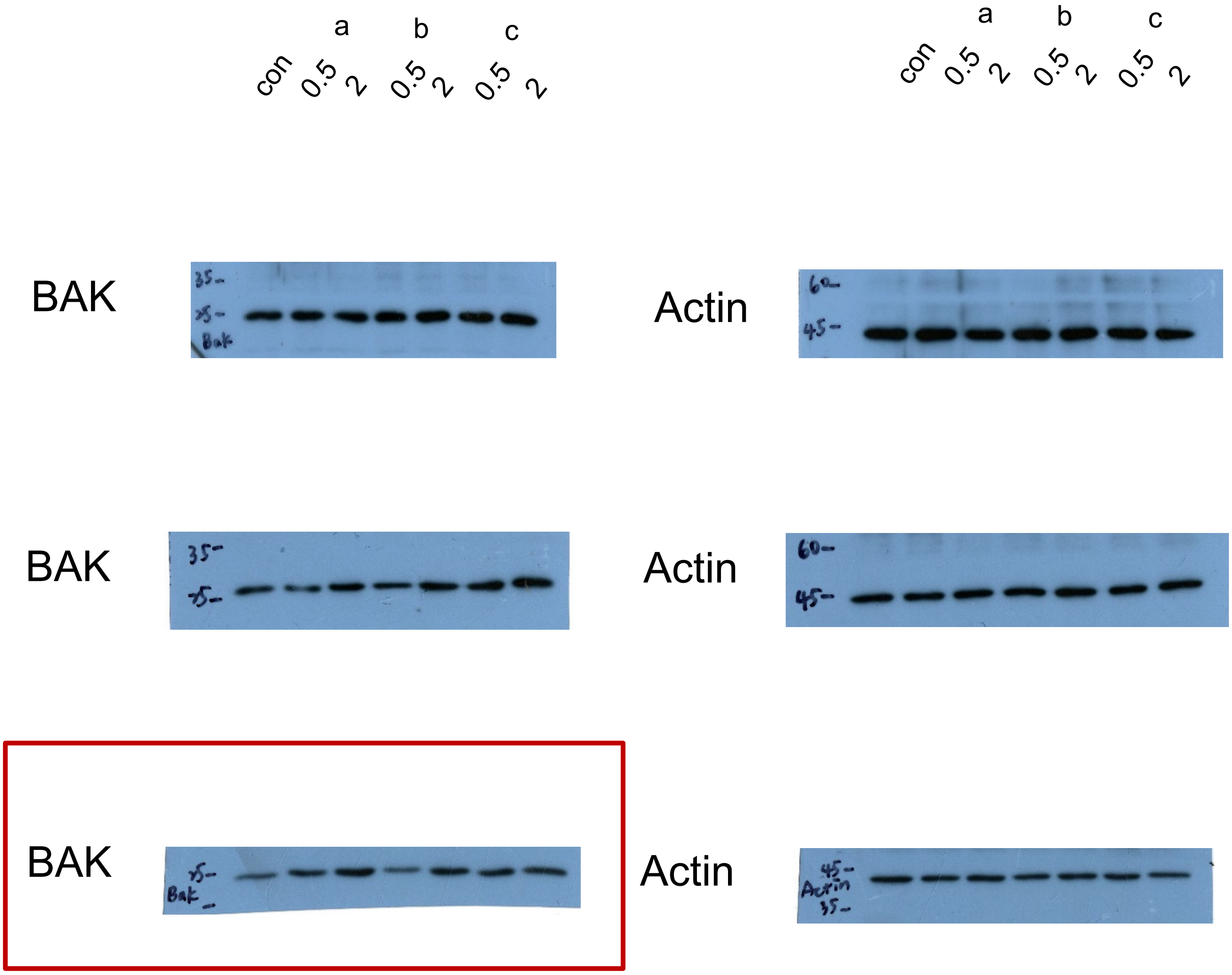

BAK	CON	a-0.5	a-2	b-0.5	b-2	c-0.5	c-2		
**1**	1	0.96	1.15	1.18	1.32	1.30	1.78		
**2**	1	0.82	1.22	0.94	1.27	1.49	1.62		
**3**	1	1.57	1.84	1.04	1.70	1.59	1.99		

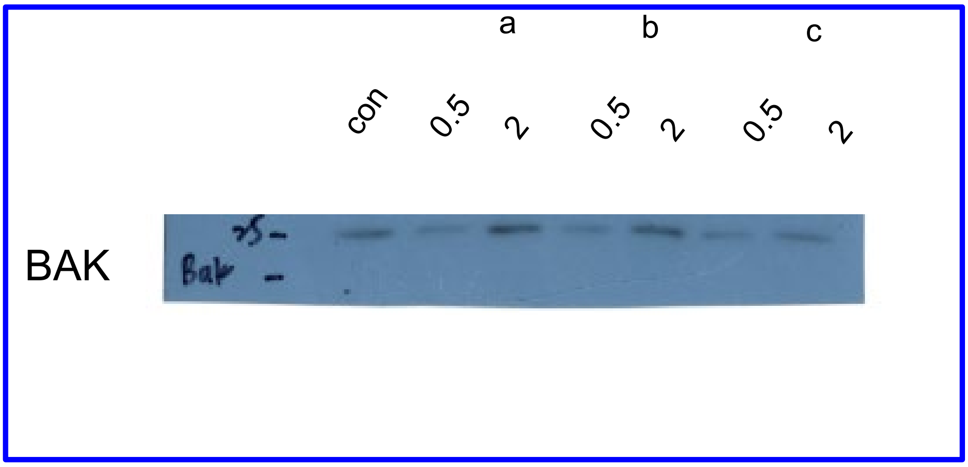
a
b
c
con
0.5
2
0.5
2
0.5
2
BAK
25
Bak

Figure 3B HCT116 p53 wild-type cells

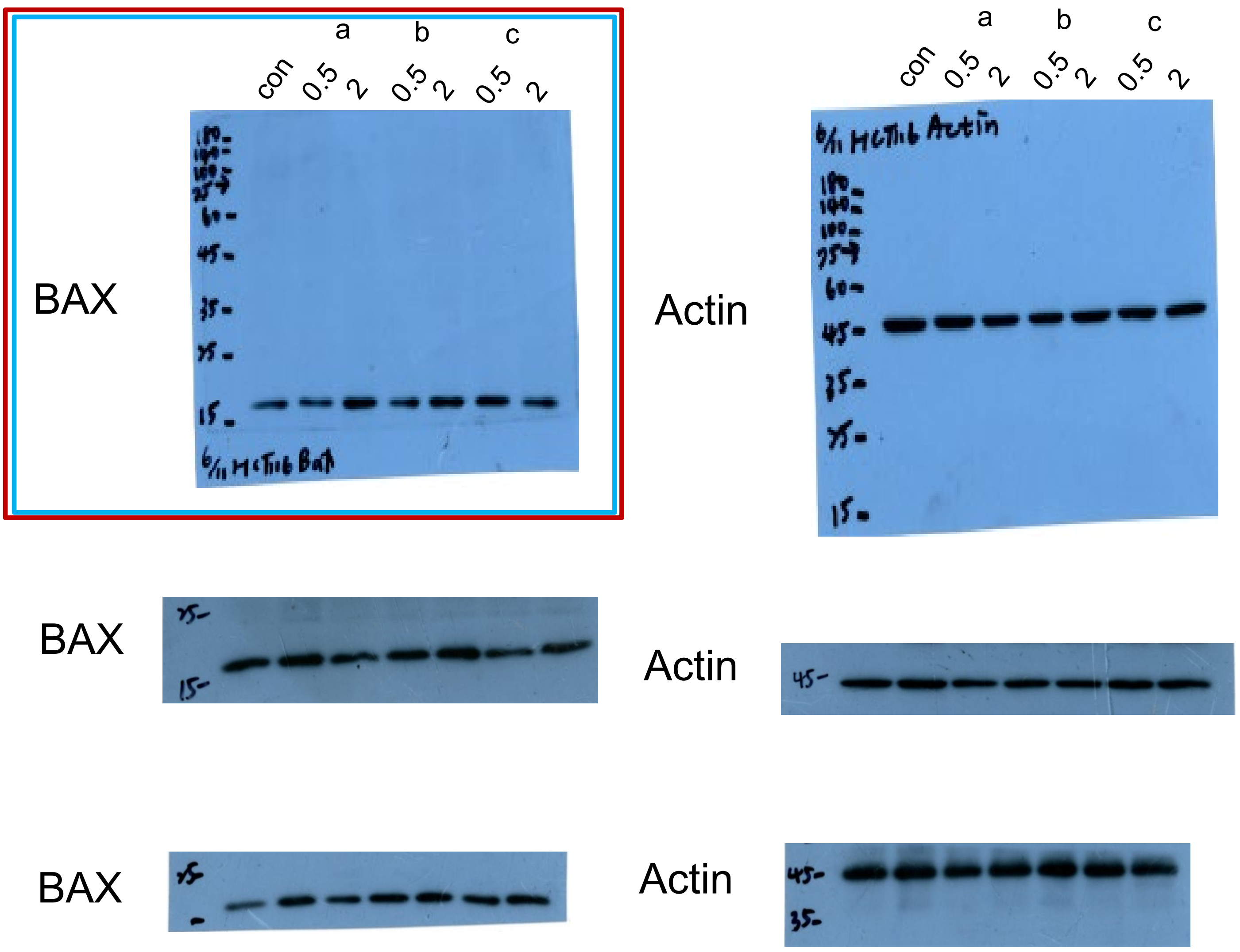

BAX	CON	a-0.5	a-2	b-0.5	b-2	c-0.5	c-2		
1	1.00	1.18	2.36	1.55	2.01	2.04	1.77		
2	1.00	1.14	1.17	1.03	1.32	0.77	0.77		
3	1.00	1.69	1.55	1.87	1.84	1.63	2.16		

Figure 3 B   HCT116 p53 wild-type cells

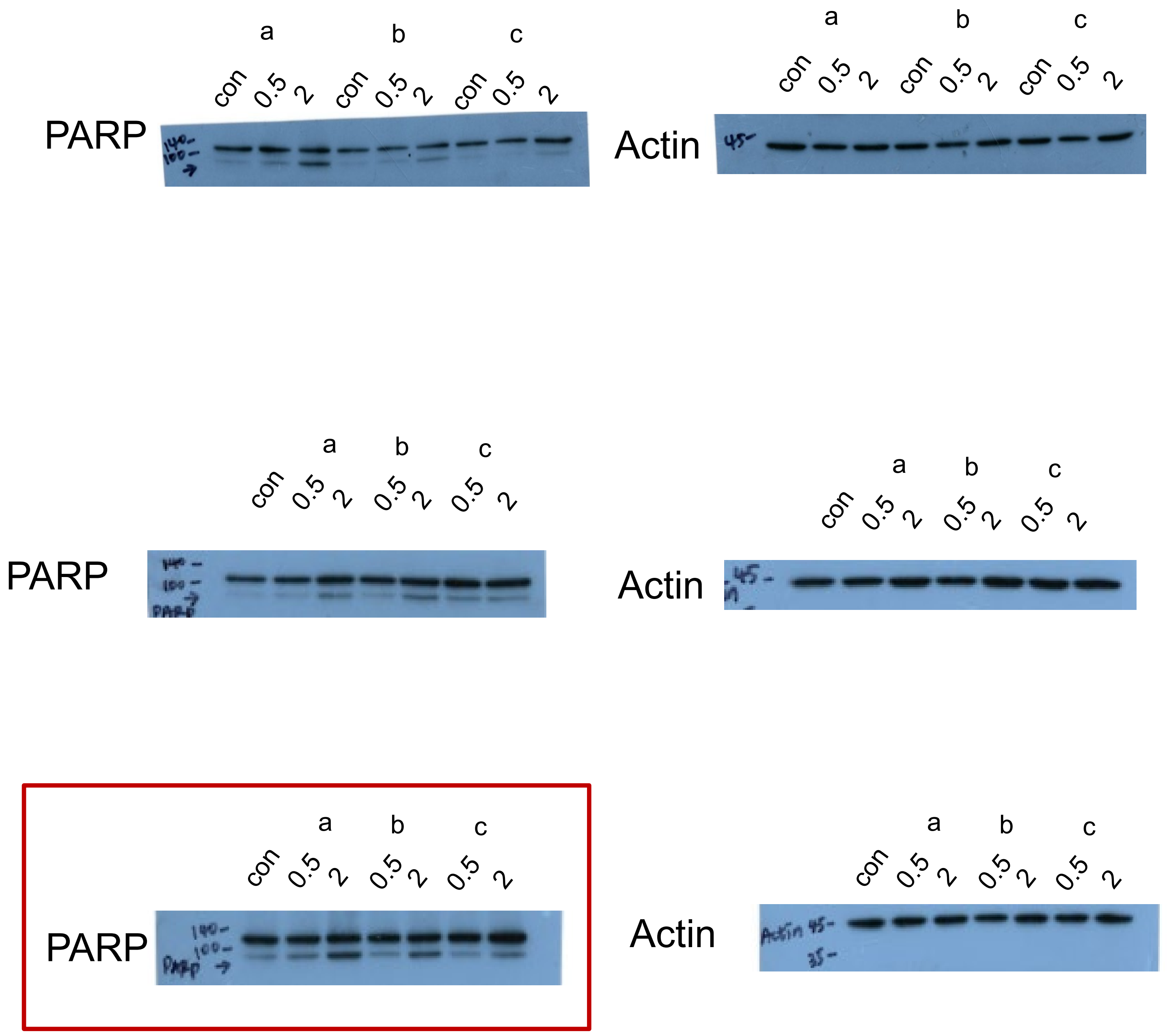

c-PARP	CON	a-0.5	a-2	b-0.5	b-2	c-0.5	c-2		
**1**	1.00	2.01	4.50	1.00	1.35	6.86	1.00		
**2**	1.00	1.59	3.59	2.34	4.45	4.00	3.86		
**3**	1.00	1.22	2.31	1.00	1.61	0.76	1.21		

Figure 3B HCT116 p53 wild-type cells

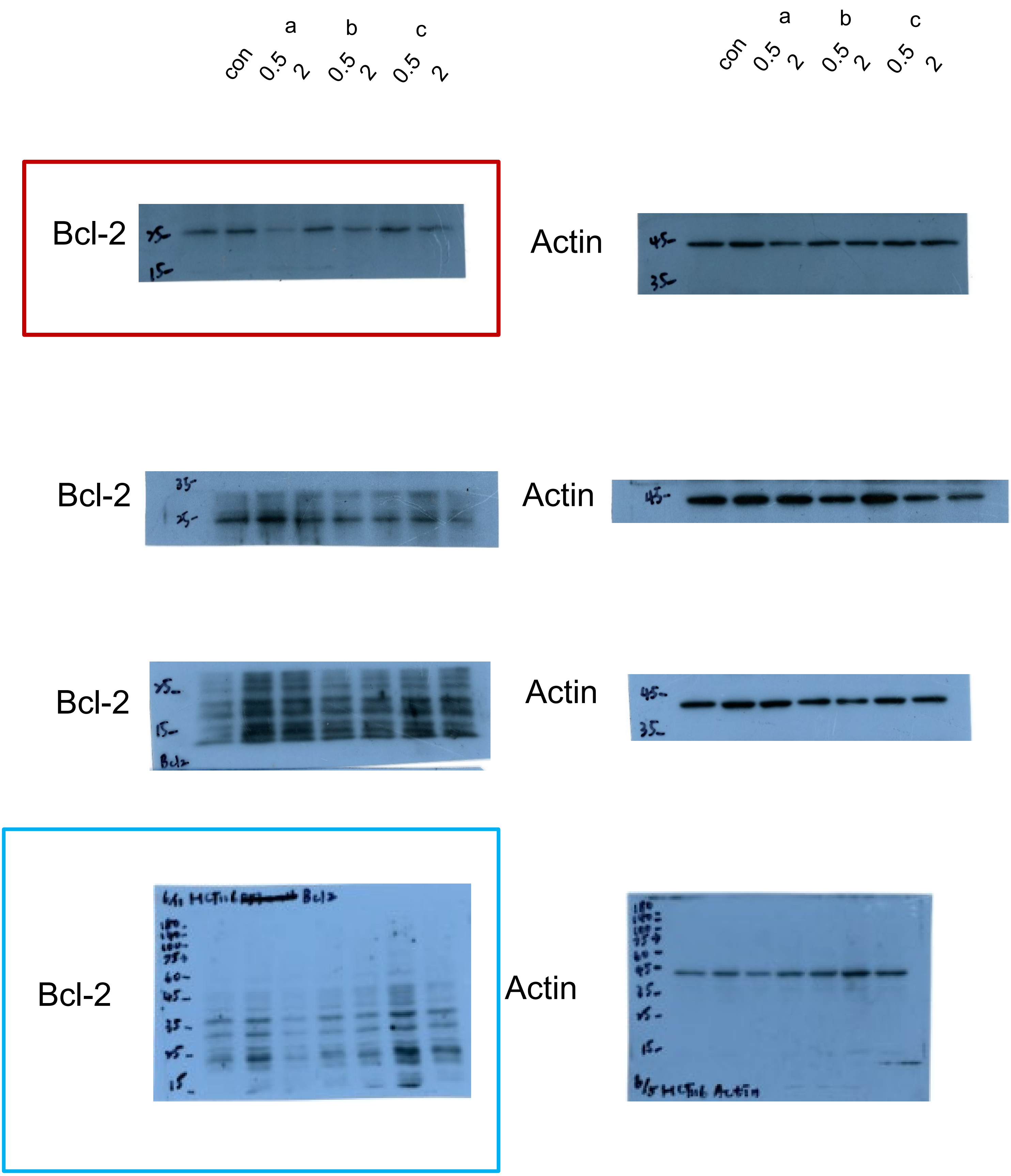

Bcl-2	CON	a-0.5	a-2	b-0.5	b-2	c-0.5	c-2		
1	1.00	0.95	0.69	1.12	0.83	1.14	0.60		
2	1.00	1.25	1.08	0.83	0.57	1.27	0.90		
3	1.00	2.56	2.48	1.90	2.43	2.29	2.50		
4	1.00	1.41	0.64	0.75	0.67	1.18	0.96		

Figure3C Cell Cycle analysis HCT116 p53 wild-type cells

Con (H_2O)

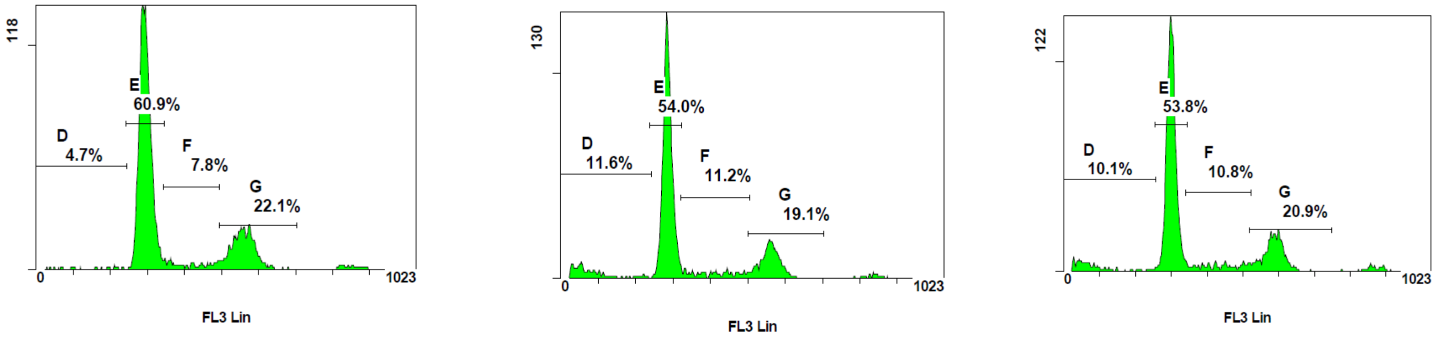

a (0.5µM)

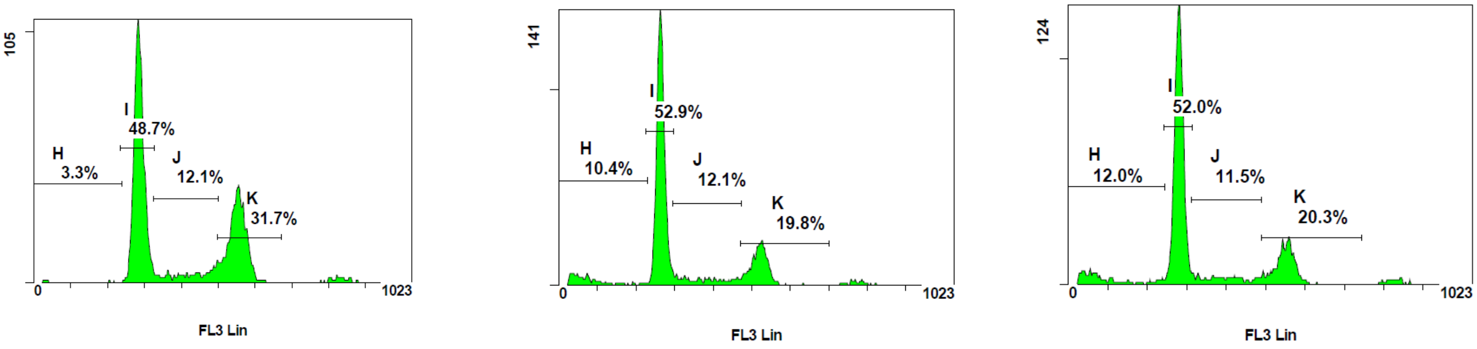

a (2µM)

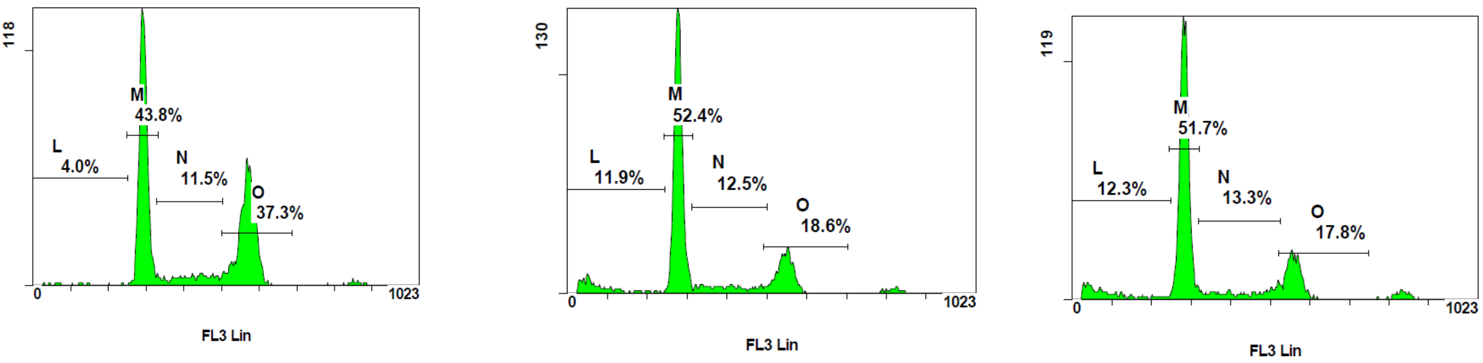

Figure3C Cell Cycle analysis HCT116 p53 wild-type cells

b (0.5µM)

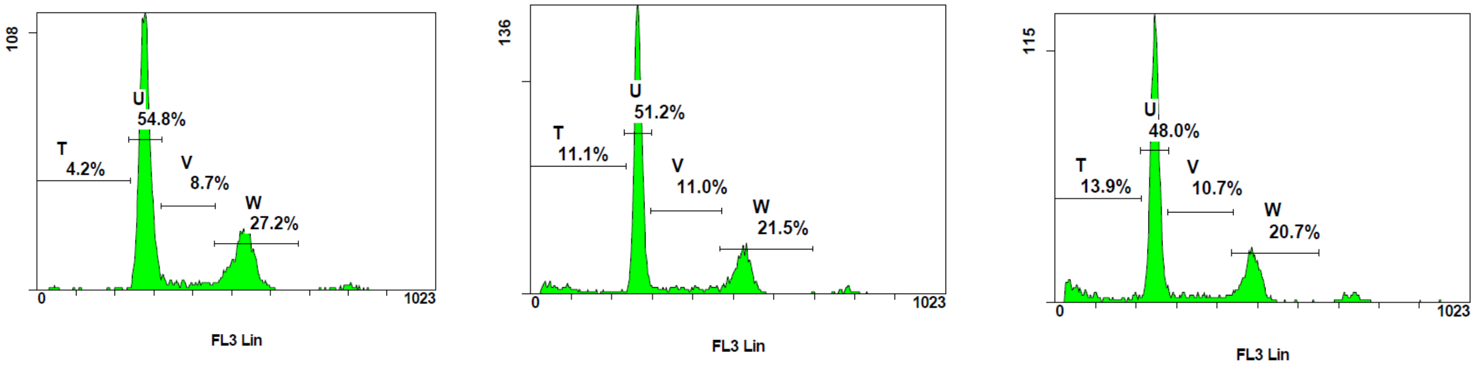

b (2µM)

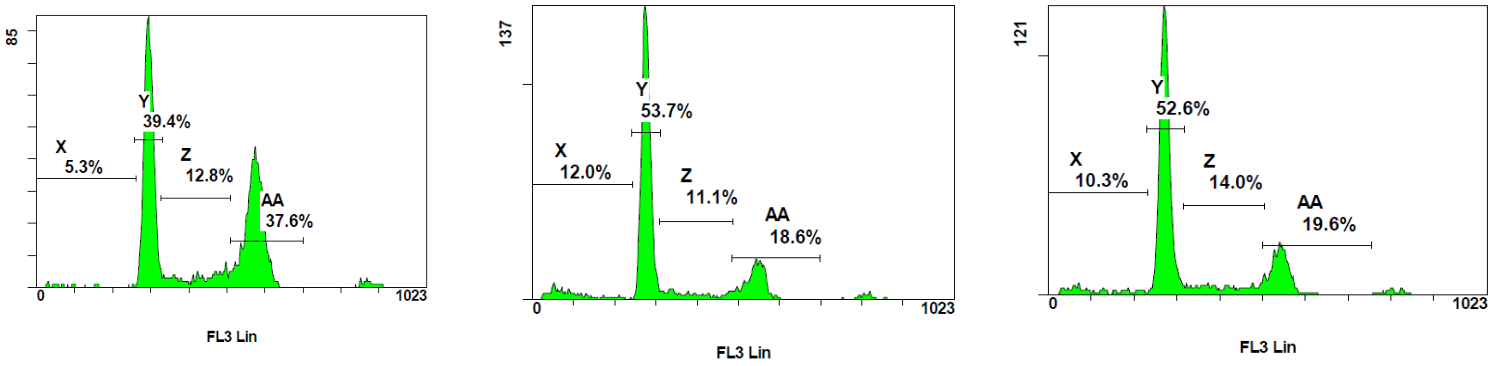

c (0.5µM)

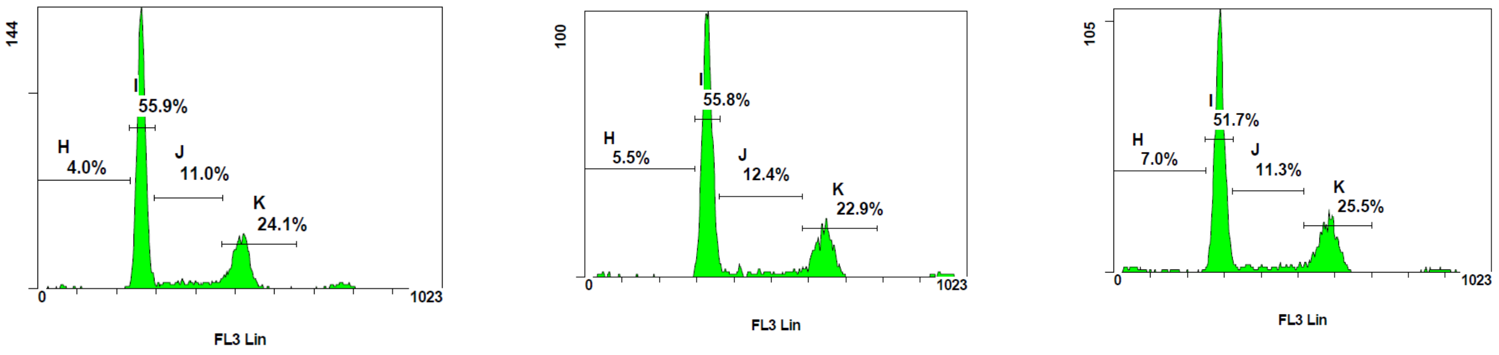

Figure3C Cell Cycle analysis HCT116 p53 wild-type cells

c (2μM)

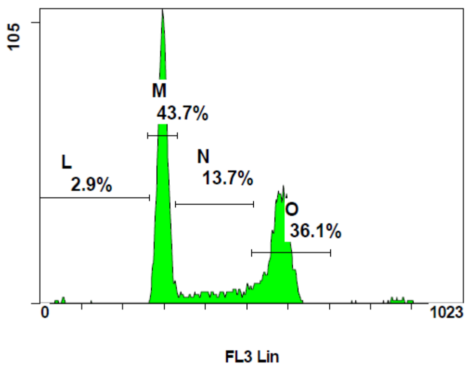

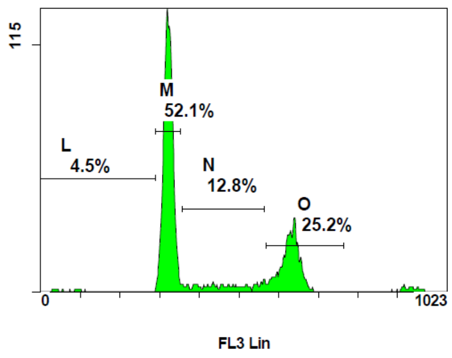

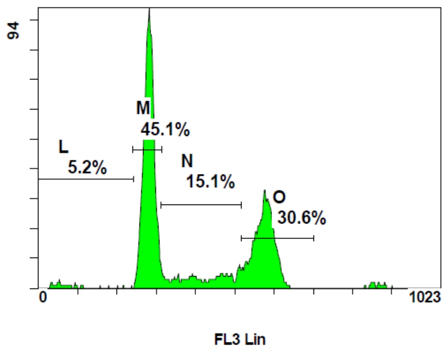

Figure 5A Apoptosis HCT116 p53-null cells

Con (H2O)

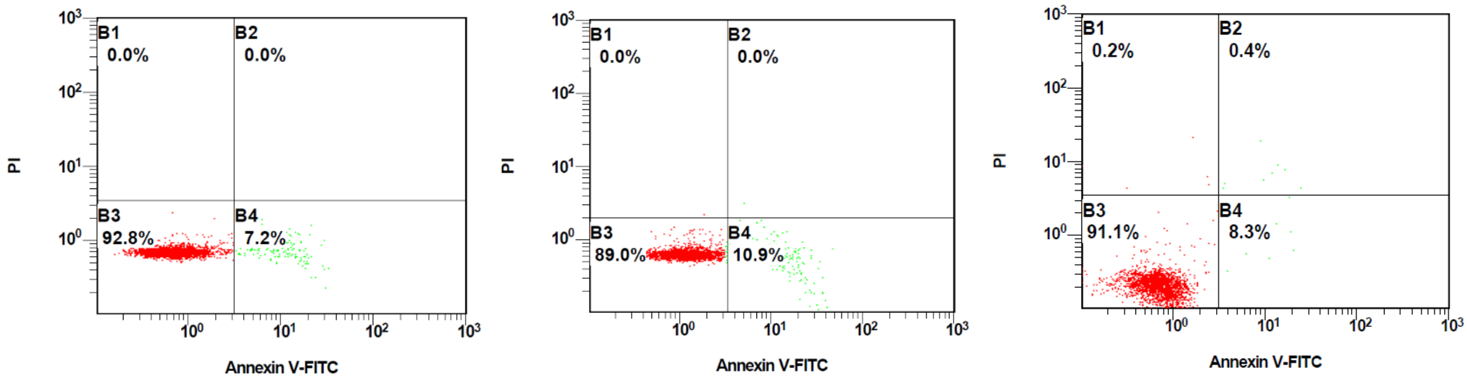

PC

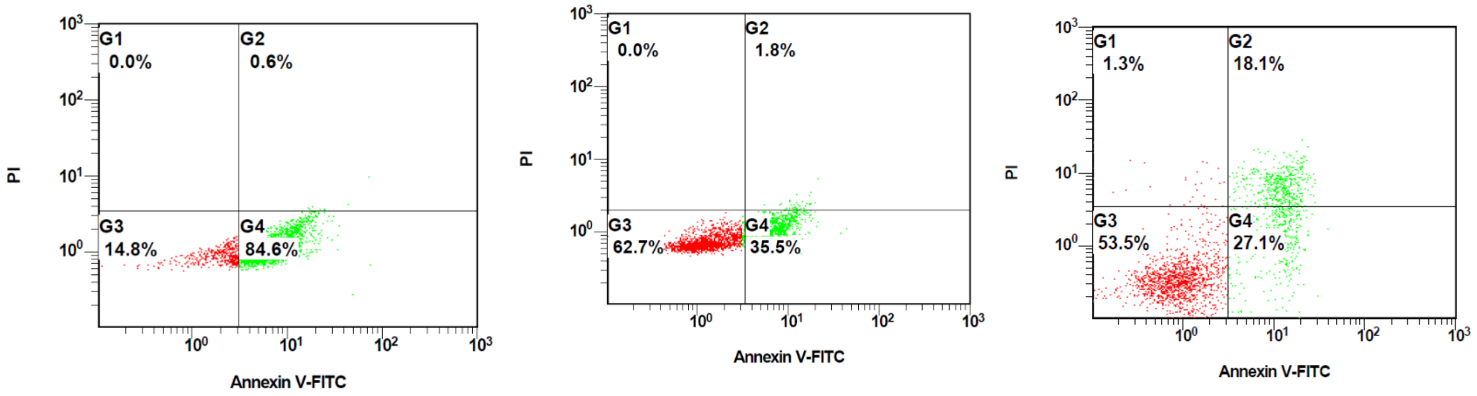

a (0.5μM)

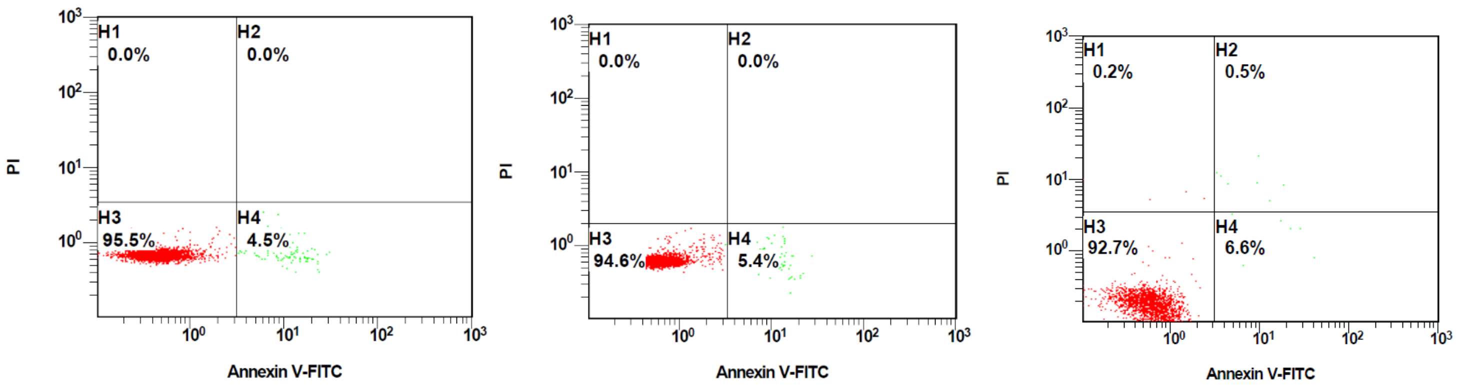

Figure 5A Apoptosis HCT116 p53-null cells

a (2μM)

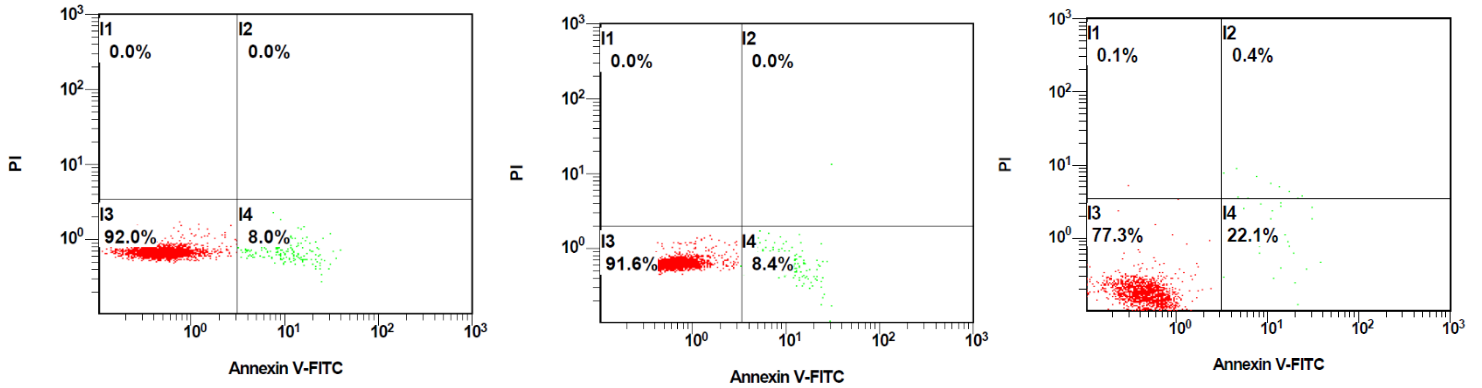

b (0.5μM)

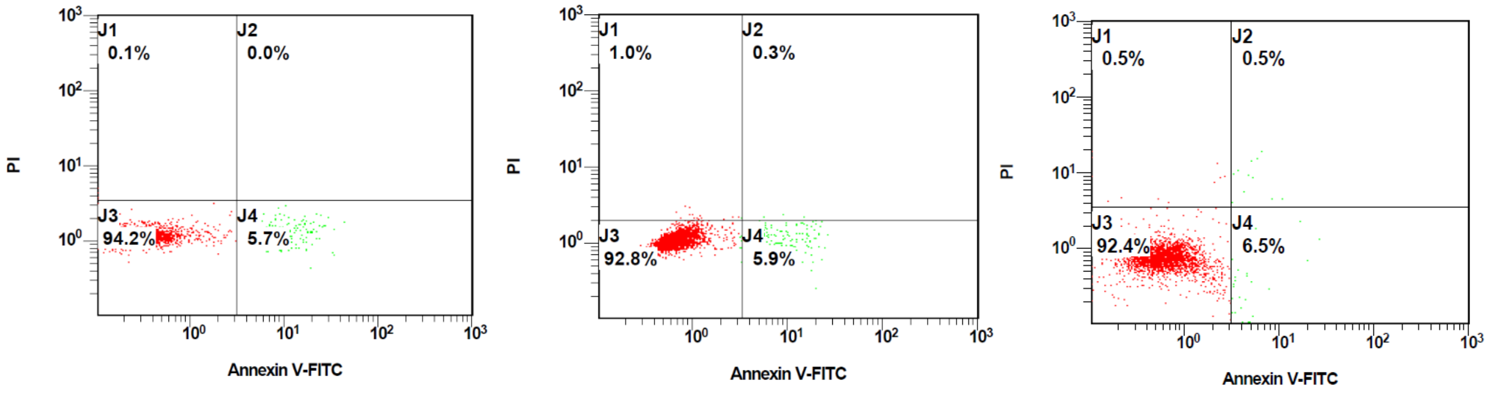

b (2μM)

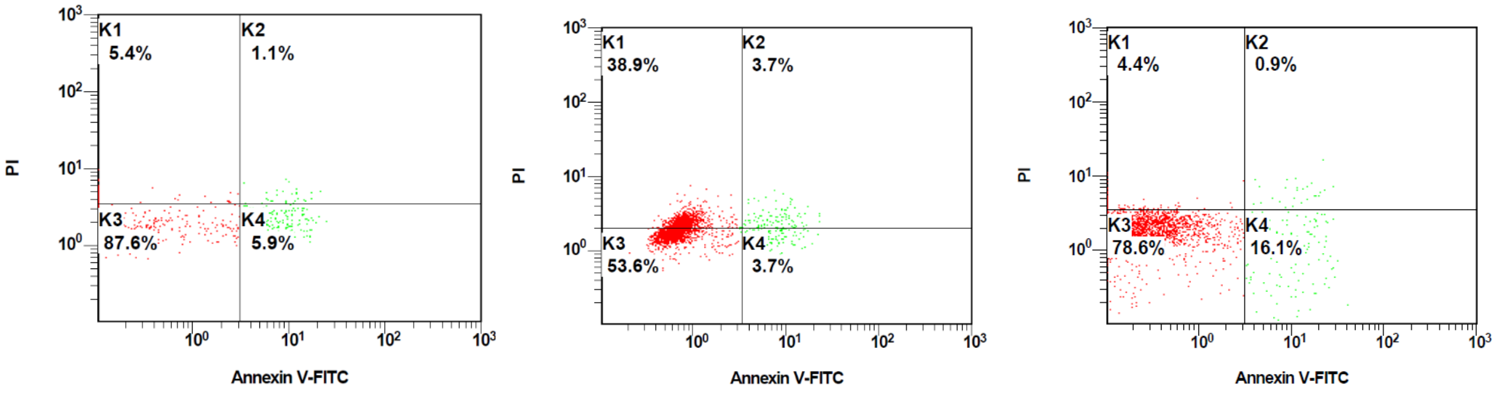

Figure 5A Apoptosis HCT116 p53-null cells

c (0.5μM)

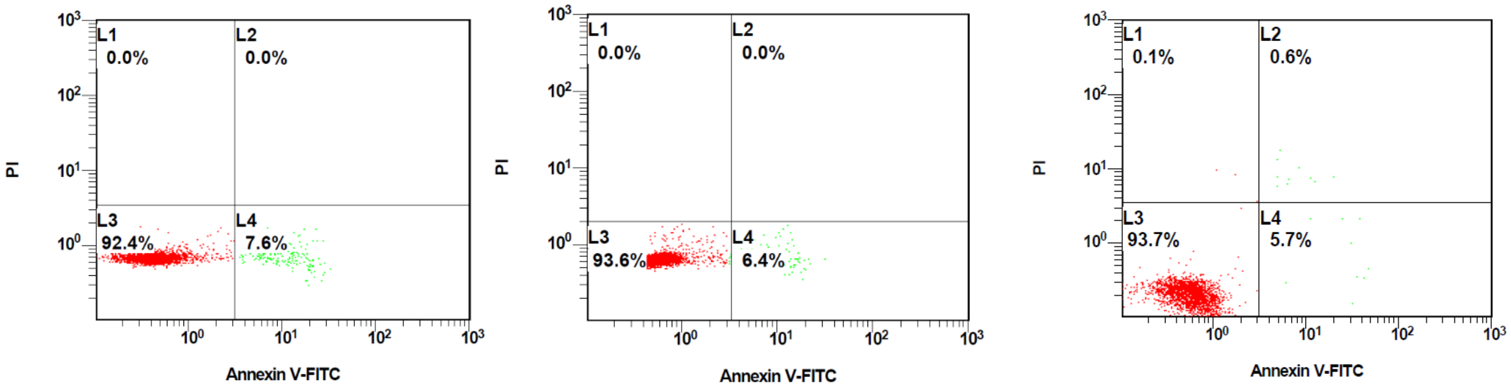

c (2μM)

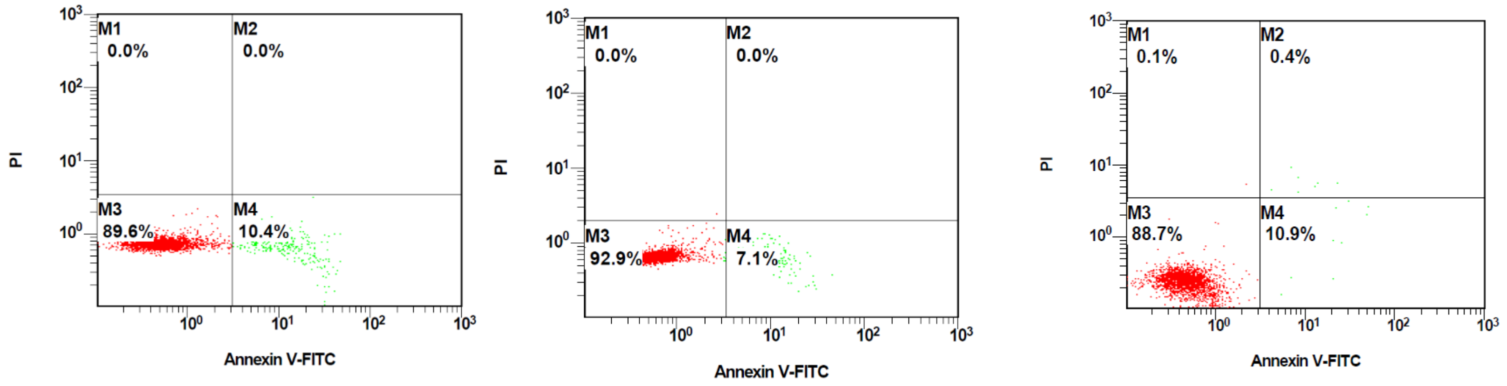

Figure 5B Western blot HCT116 p53-null cells

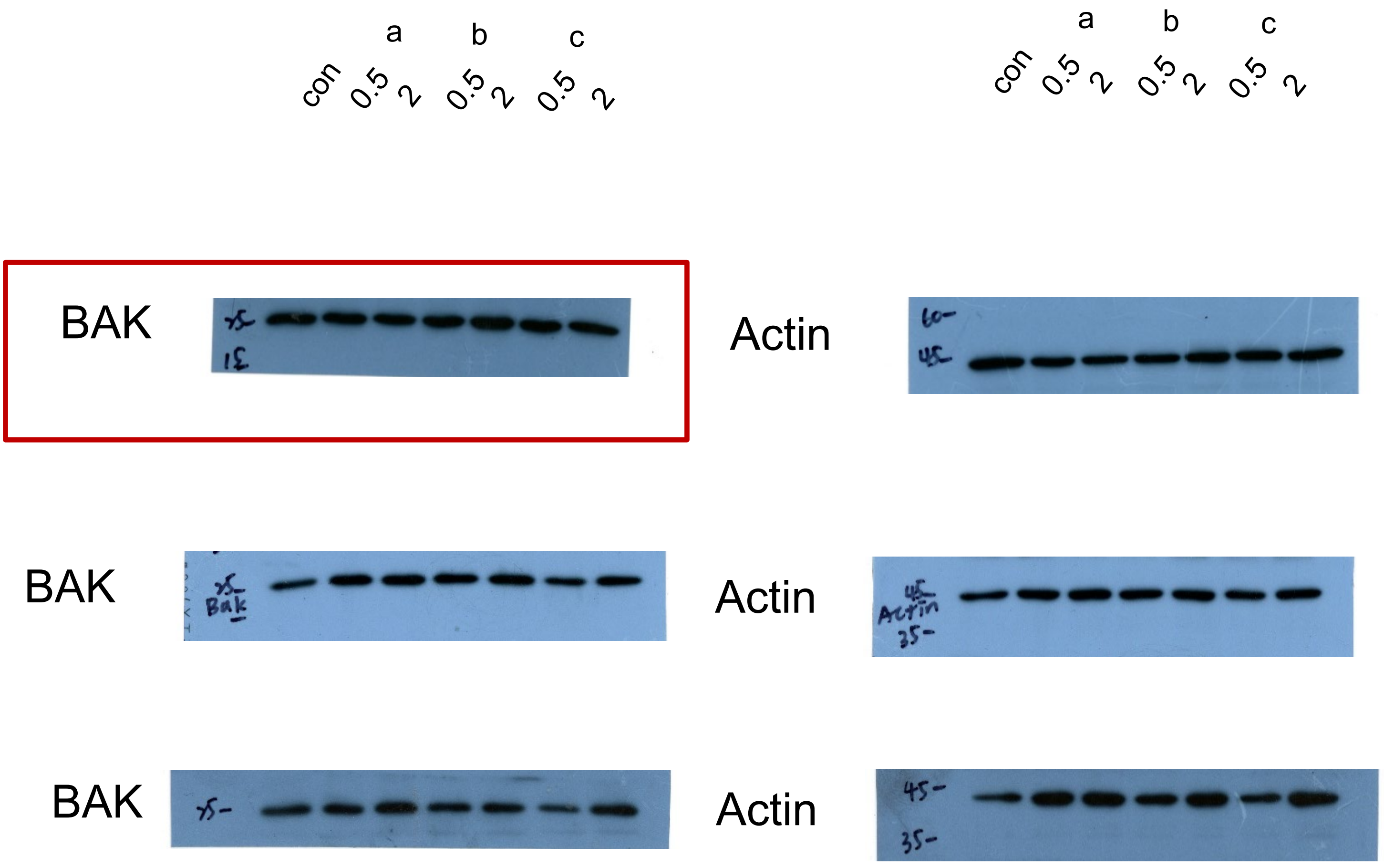

BAK	CON	a-0.5	a-2	b-0.5	b-2	c-0.5	c-2		
1	1.00	1.36	1.31	1.32	1.27	1.15	1.06		
2	1.00	1.10	1.00	1.09	1.16	1.01	0.98		
3	1.00	0.57	0.64	0.71	0.55	0.56	0.60		

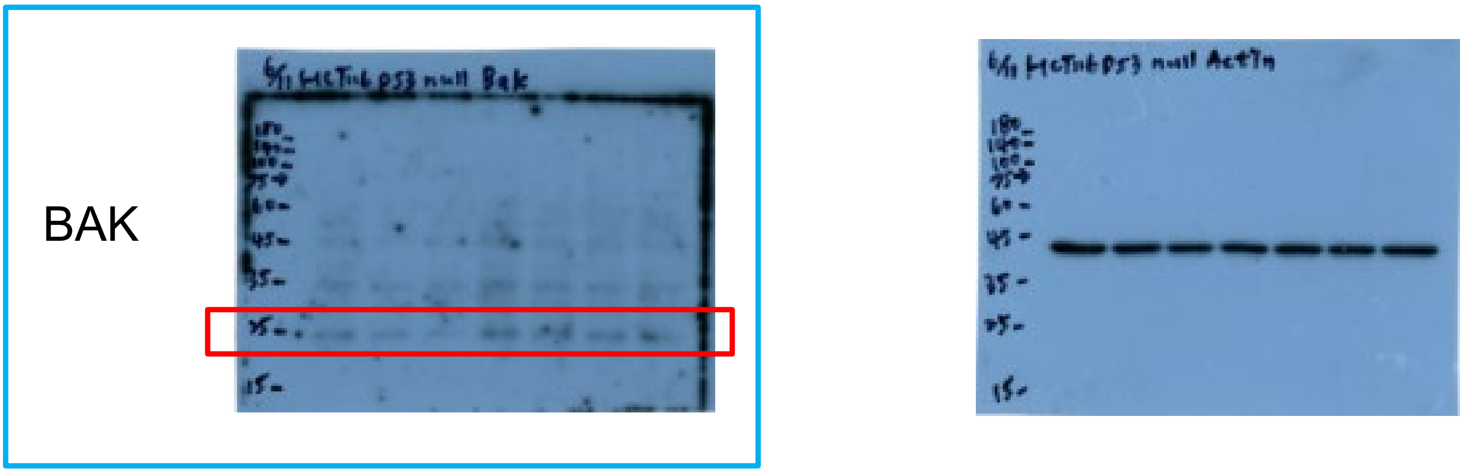

Figure 5B HCT116 p53-null cells

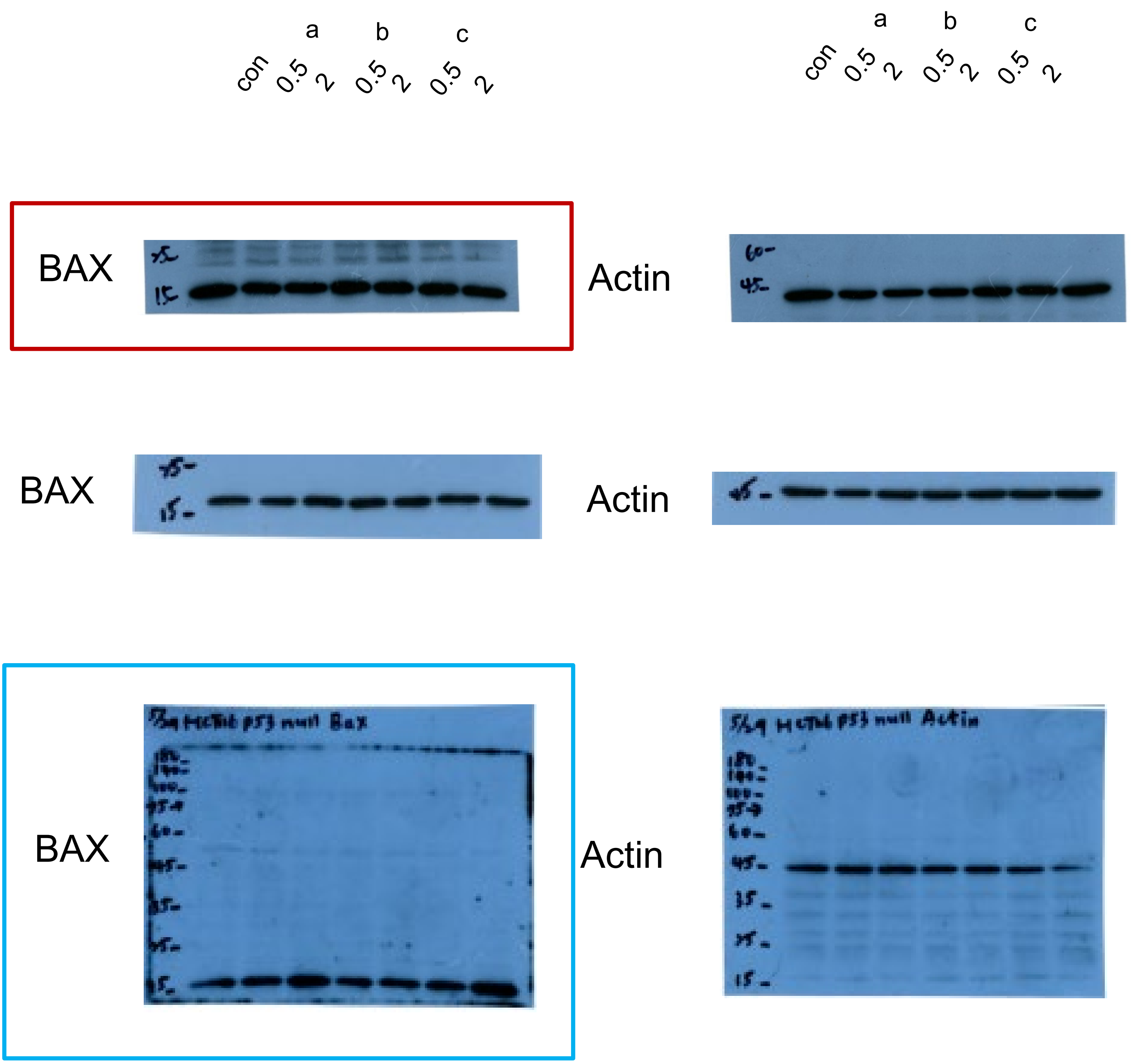

BAX	CON	a-0.5	a-2	b-0.5	b-2	c-0.5	c-2		
1	1.00	1.10	1.19	1.14	0.88	0.95	0.71		
2	1.00	1.23	1.30	1.24	1.22	1.18	1.00		
3	1.00	1.08	1.57	1.16	1.23	1.49	3.39		

Figure 5B HCT116 p53-null cells

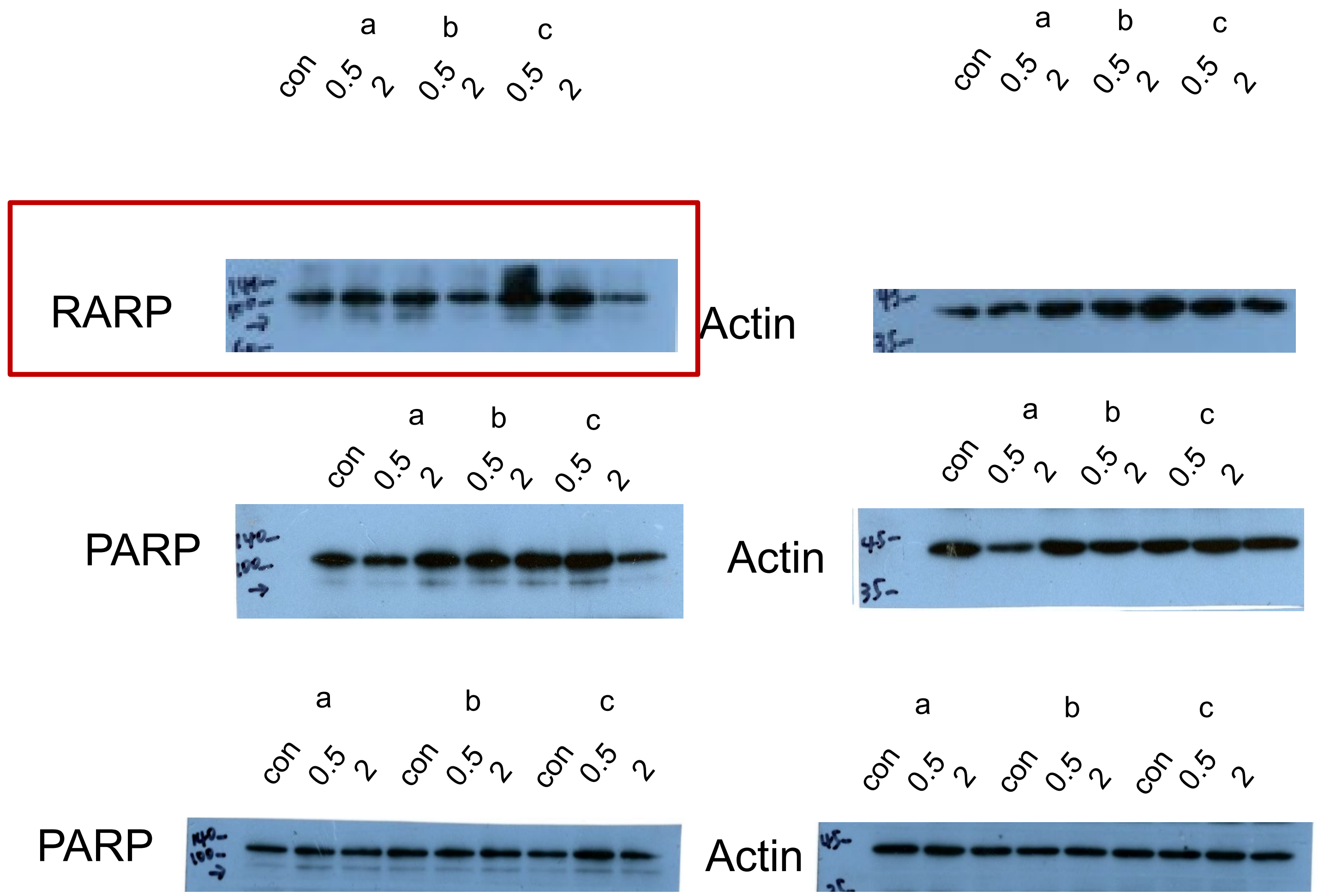

c-PARP	CON	a-0.5	a-2	b-0.5	b-2	c-0.5	c-2		
1	1.00	1.47	1.09	0.31	0.82	1.03	0.33		
2	1.00	1.44	2.20	2.34	2.34	2.92	0.47		
	CON	a-0.5	a-2	CON	b-0.5	b-2	CON	c-0.5	c-2
3	1.00	1.99	1.79	1.00	0.90	0.94	1.00	2.19	1.98

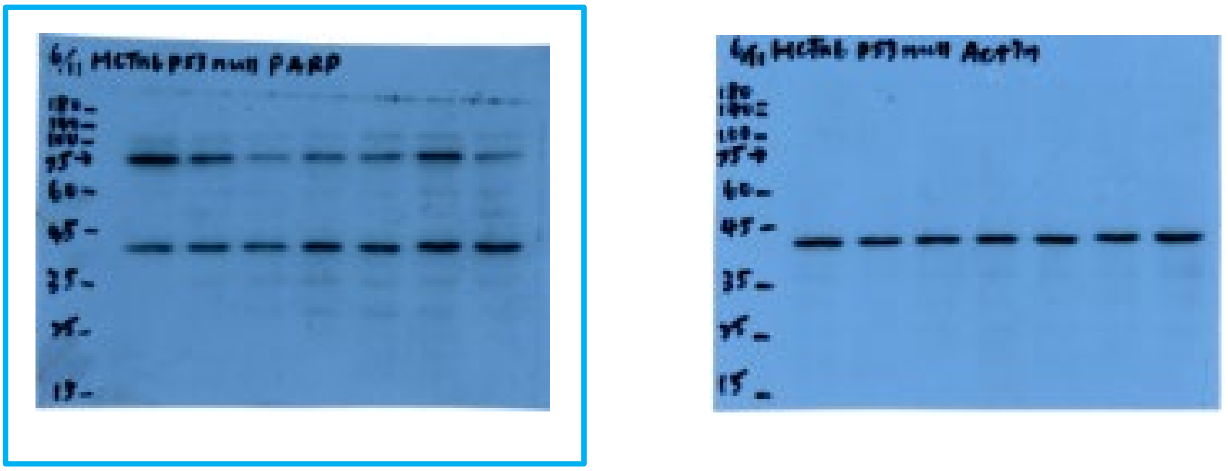

Figure 5B HCT116 p53-null cells

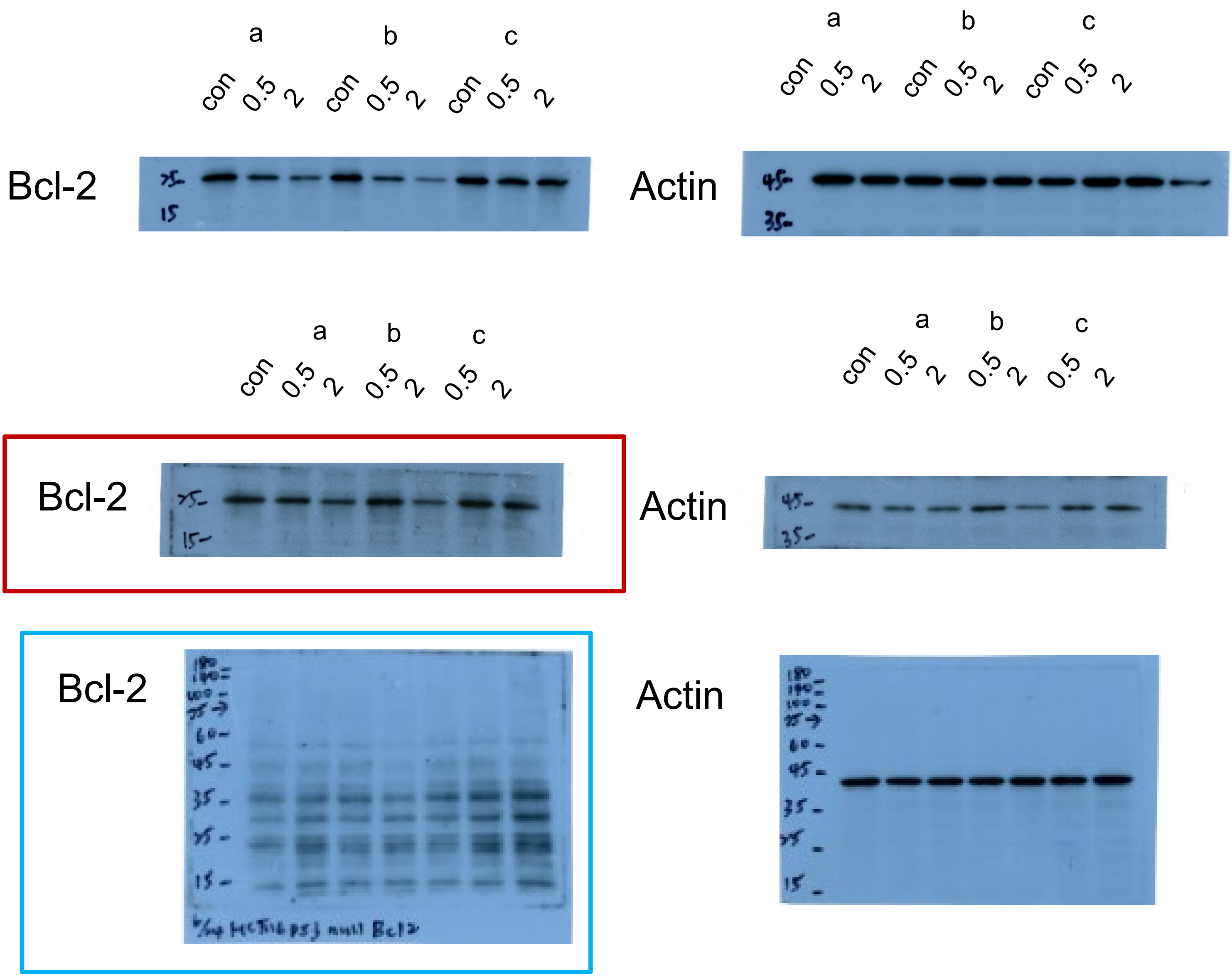

Bcl-2	CON	a-0.5	a-2	CON	b-0.5	b-2	CON	c-0.5	c-2
**1**	1.00	0.66	0.51	1.00	0.53	0.38	1.00	0.92	2.11
	**CON**	**a-0.5**	**a-2**	**b-0.5**	**b-2**	**c-0.5**	**c-2**		
**2**	1.00	1.29	1.04	1.25	1.03	1.05	0.94		
**3**	1.00	1.88	1.22	1.38	1.09	1.90	2.29		

Figure 5C Cell Cycle analysis HCT116 p53-null cells

Con (H2O)

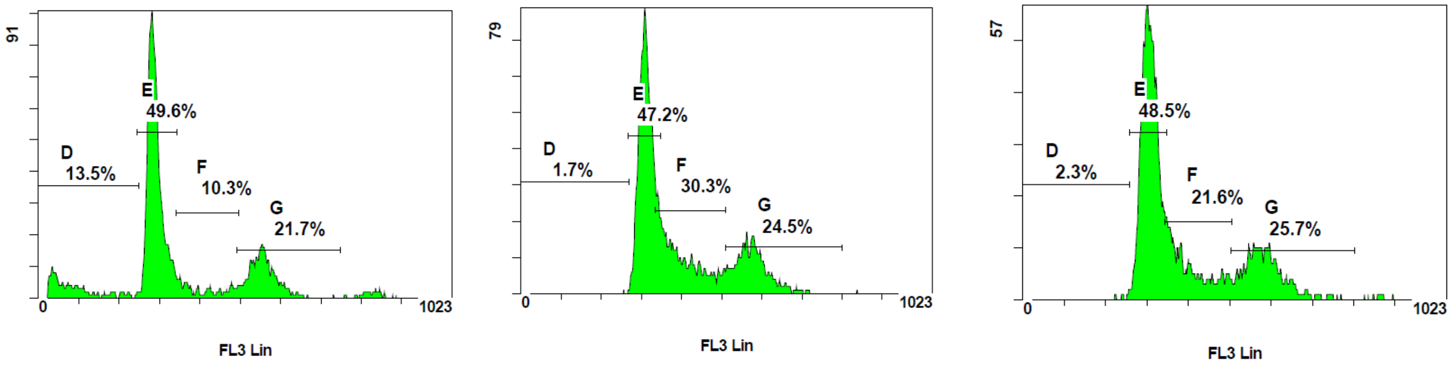

a (0.5µM)

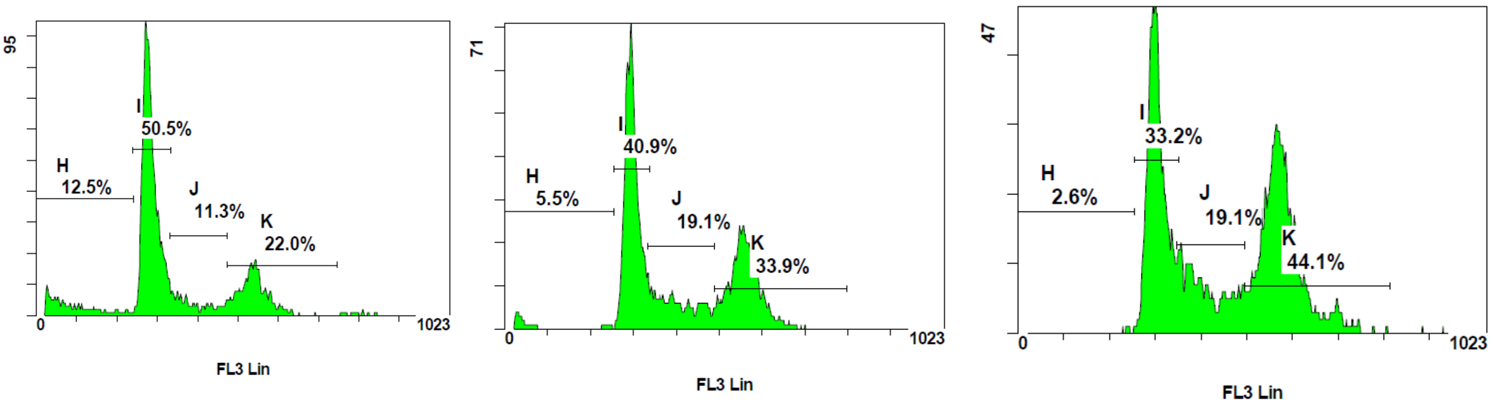

a (2µM)

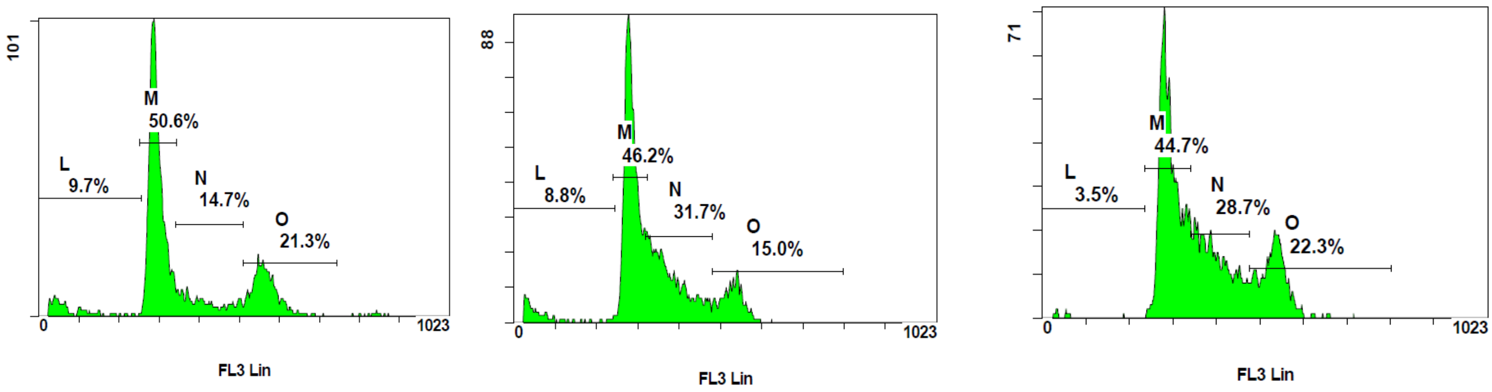

Figure 5C Cell Cycle analysis HCT116 p53-null cells

b (0.5µM)

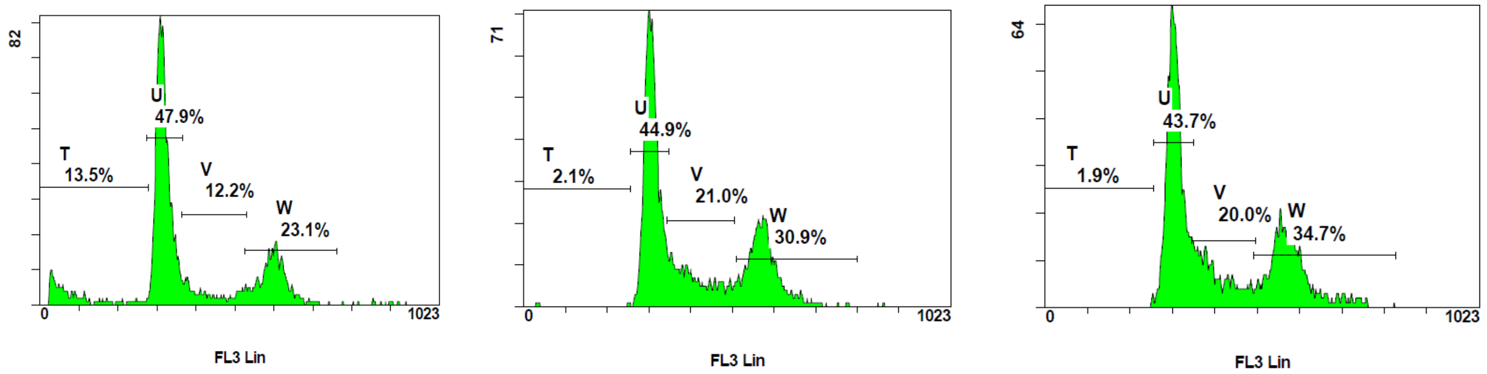

b (2µM)

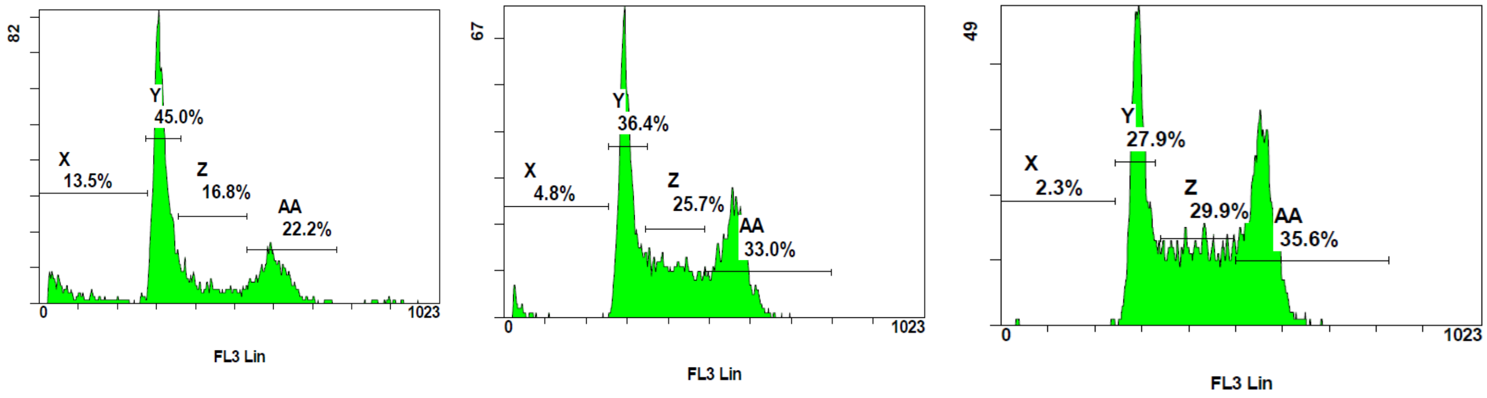

c (0.5µM)

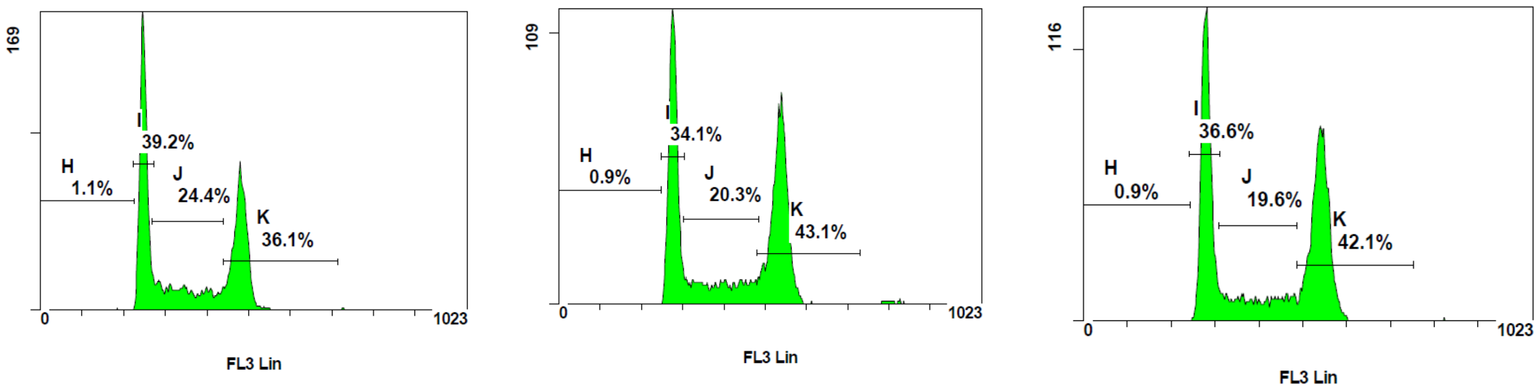

Figure 5C Cell Cycle analysis
HCT116 p53-null cells

c (2µM)

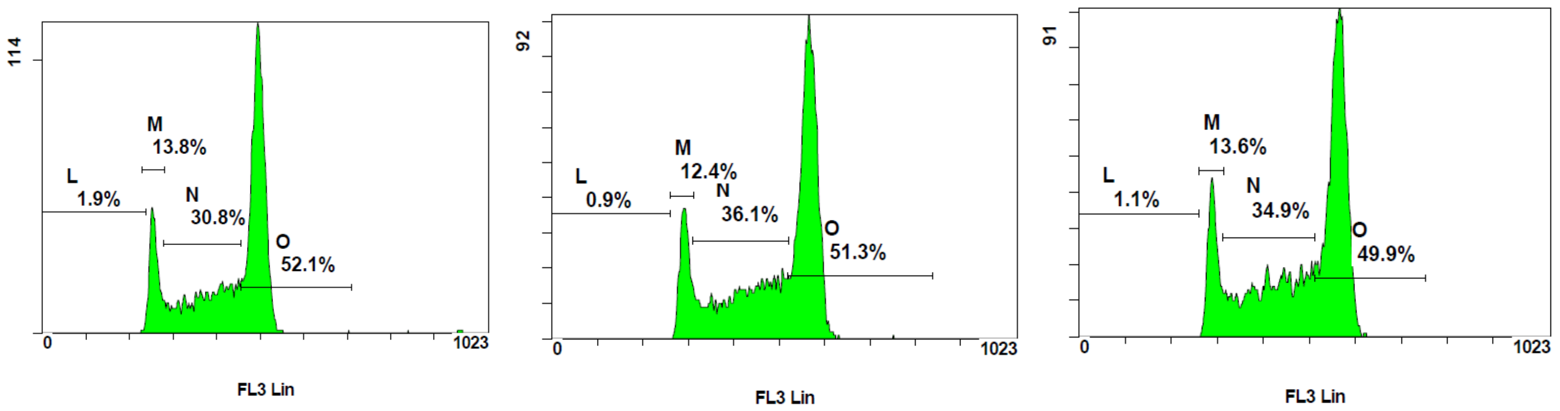

Figure 6B HCT116 p53 wild-type cells

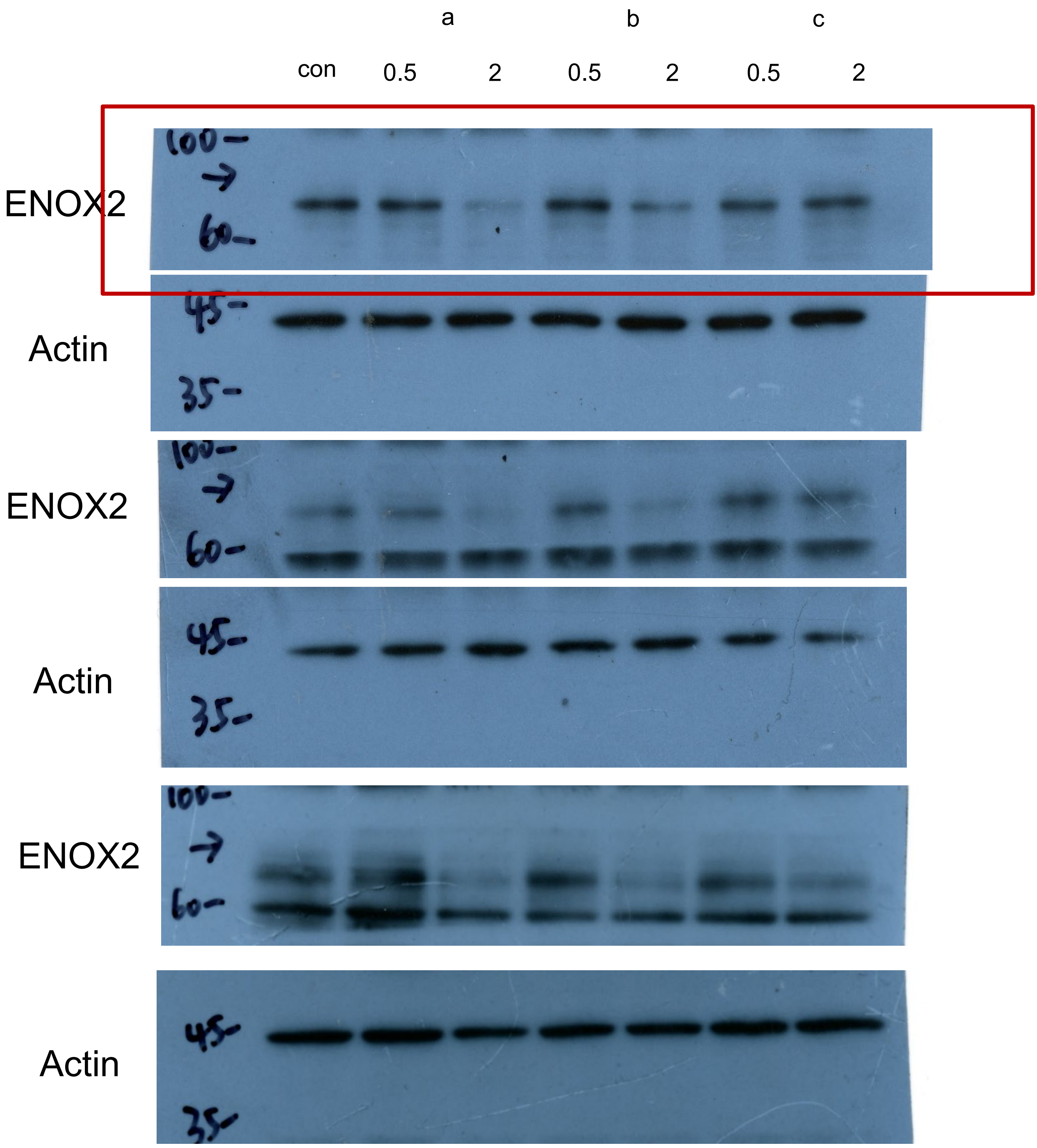

ENOX2	CON	a-0.5	a-2	b-0.5	b-2	c-0.5	c-2		
1	1.00	1.00	0.30	1.21	0.46	0.83	0.95		
2	1.00	0.97	0.42	1.06	0.43	1.67	1.81		
3	1.00	1.15	0.62	1.09	0.56	0.95	0.58		

Figure 6B HCT116 p53 wild-type cells

ENOX2

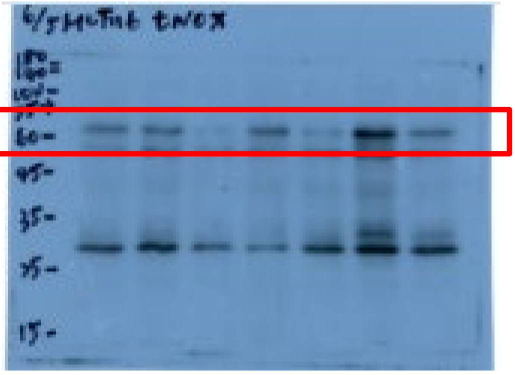

Actin

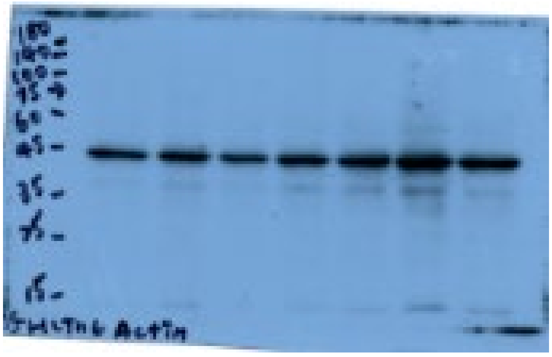

Figure 6B HCT116 p53-null cells

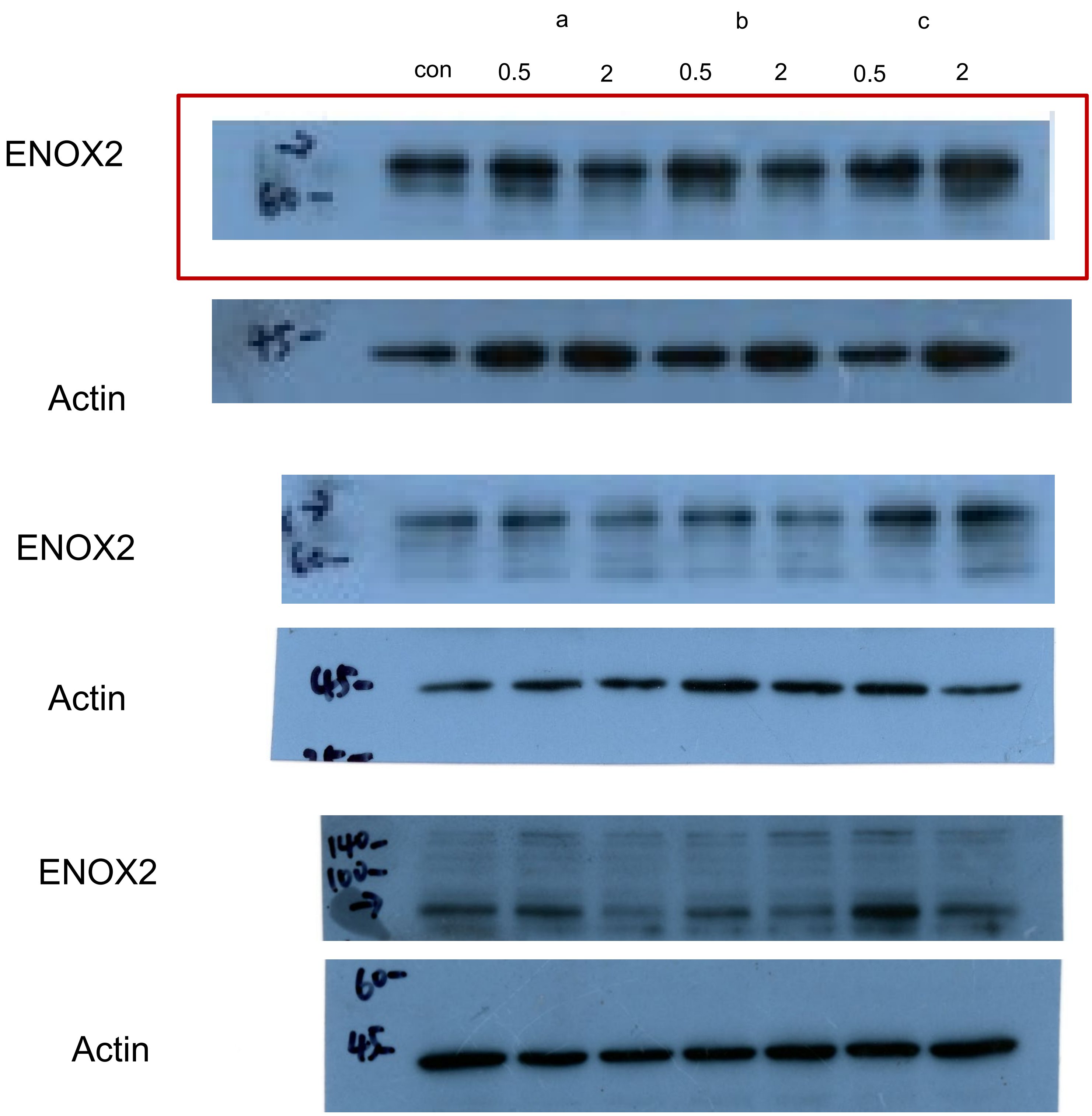

ENOX2	CON	a-0.5	a-2	b-0.5	b-2	c-0.5	c-2		
1	1.00	0.68	0.51	0.84	0.49	1.08	0.75		
2	1.00	0.87	0.74	0.61	0.49	0.97	1.61		
3	1.00	1.26	0.64	0.92	0.73	1.61	0.97		

Figure 6B HCT116 p53-null cells

ENOX2

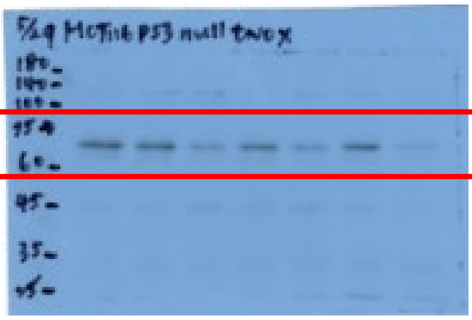

Actin

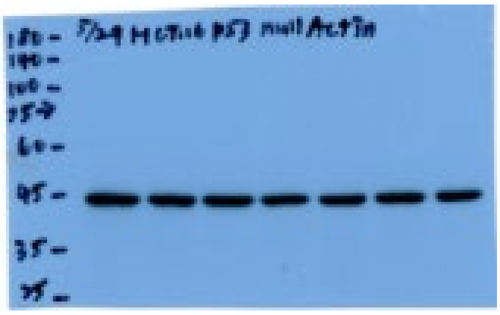

Supplement: Supplementary file 1 [file biomolecules-16-01043-s001.zip › biomolecules-4404228-supplementary.pdf]
